# AgrOmicSo: A client-server interface for accessible large-scale analysis of next-generation sequencing data

**DOI:** 10.1371/journal.pone.0348571

**Published:** 2026-06-01

**Authors:** Dong-Jun Lee, Tae-Ho Lee, Taesoo Kwon

**Affiliations:** 1 Supercomputing Center, National Institute of Agricultural Science, Jeonju, Republic of Korea; 2 Corporate R&D Center, Cloud9, Cheongju-si, Republic of Korea; University of the West of Scotland, UNITED KINGDOM OF GREAT BRITAIN AND NORTHERN IRELAND

## Abstract

The analysis of large-scale next-generation sequencing (NGS) data requires substantial computational power, often necessitating the use of high-performance computing (HPC) environments. However, the command-line interfaces for these resources create a significant barrier for many researchers. To bridge this gap, we developed AgrOmicSo (Agri-bio Omics Solution), a software solution designed as a user-friendly interface to a powerful server-side analysis engine. AgrOmicSo’s client-server architecture allows researchers to manage and execute complex, large-scale NGS data analysis pipelines on a remote server directly from an intuitive graphical user interface on their local computer. The software integrates a comprehensive suite of bioinformatics tools for quality control, read mapping, variant calling, and annotation. Notably, it supports three distinct variant calling algorithms—GATK, DeepVariant, and VarScan—offering users flexibility for their specific research needs. AgrOmicSo provides both a “One-Step” mode for rapid, automated batch processing and a “Step-by-Step” mode for detailed, customized analyses. This paper describes the architecture, implementation, and utility of AgrOmicSo as an interface for large-scale genomic analysis, highlighting its potential to advance research by making powerful computational resources more accessible, efficient, and reproducible for a broader scientific community. The client and server program of AgrOmicSo are freely available at https://agromicso.com.

## Introduction

Currently, massive parallel sequencing has become an important tool in many biological research fields such as cancer genomics, rare diseases, and even crop breeding, and it is changing modern biological research into data science [1]. Thus, large amounts of sequencing data are constantly being produced, and the need for rapid and efficient analysis of data has increased. While the computational power of personal computers (PCs) has increased, analyzing large NGS datasets locally can be inefficient due to the need to install, configure, and manage a multitude of bioinformatics tools and their dependencies [2]. The process of identifying genetic variants from these large datasets is a critical step, but it is often intricate, repetitive, and demands significant concentration from the researcher.

High-performance computing (HPC) environments and supercomputers offer the necessary resources for handling large-scale genomic data. However, it is difficult for researchers without bioinformatics knowledge to analyze large amounts of sequencing data using servers that are mostly operated on Unix/Linux. This often creates a bottleneck, hindering the timely analysis and interpretation of valuable sequencing data. Consequently, a tool that allows researchers unfamiliar with bioinformatics analysis to easily analyze their sequencing data using a server is necessary [3].

Several platforms have been developed to address this challenge by providing a more accessible interface to complex bioinformatics tools (Table 1). Web-based platforms like Galaxy [4] provide a graphical interface for building and running workflows, but they require significant administrative effort for installation and maintenance, and public instances may have performance limitations for large-scale jobs. Commercial solutions such as CLC Genomics Server, Seven Bridges, and DNAnexus offer robust, enterprise-level client-server capabilities, but they can be prohibitively expensive and heavily reliant on cloud infrastructure, which places them out of reach for many academic labs. Additionally, platforms like NanoForms (https://pubmed.ncbi.nlm.nih.gov/35368340/), which is tailored for Oxford Nanopore microbial genome analysis, demonstrate the growing diversity in interface and deployment strategies. However, these tools are often limited by cost, platform specificity, or dependency on internet access and external servers. In contrast, many powerful open-source tools like GATK [5], DeepVariant [6], and VarScan [7] remain primarily command-line driven. While some command-line pipelines like ngs_backbone [8] simplify execution, they still require familiarity with shell scripting and the terminal environment, limiting their use by non-specialists.

**Table 1:** Comparison of platforms for NGS data analysis.

Feature	AgrOmicSo	CLC Genomics Server	DNA Nexus	Galaxy	NanoForms	Seven Bridges Platform
**Architecture**	Client-Server	Client-Server	Cloud-based (SaaS)	Web-based Server	Web-based Server	Cloud-based (SaaS)
**User Interface**	Desktop GUI	Desktop GUI	Web GUI	Web GUI	Web GUI	Web GUI
**Primary Goal**	Provide an easy interface to a dedicated server for genomic analysis	Provide a premium, enterprise-grade analysis environment	Provide a secure, scalable, and compliant cloud-based ecosystem for the management and large-scale analysis of multi-omic data	Provide a comprehensive, flexible web platform for various bioinformatics tasks	Provide a streamlined, integrated web platform for the comprehensive processing, assembly, and annotation of Oxford Nanopore prokaryotic genomes	Provide a collaborative, cloud-based platform for large-scale analysis
**Pros**	Free, open-source; Simple client setup; Direct control over a dedicated server; Supports multiple variant callers	Polished UI; Excellent support; Validated workflows	Delivers enterprise-grade scalability and industry-leading security compliance, enabling seamless global collaboration on massive datasets	Huge tool repository; Strong community support; Reproducible workflows	Offers a specialized and user-friendly integrated pipeline that automates the entire ‘raw data-to-annotated genome’ workflow for Nanopore sequencing without requiring complex bioinformatics expertise	Highly scalable; Collaborative features; CWL/WDL support
**Cons**	Requires user to set up and maintain their own server	Commercial license required; Can be a “black box”	Involves high operational costs and a significant technical learning curve, making it less accessible for individual researchers or small labs with limited budgets	Can be slow for many users on a public instance; Server admin is complex	Constrained by a 15 Mb genome size limit and a fixed selection of tools, which restricts its application to small prokaryotes and limits flexibility for highly customized research needs	Subscription-based cost; Data transfer costs can be high
**License**	Free (Open Source)	Commercial	Commercial	Free (Open Source)	Free (Open Source)/ Academic Use	Commercial (Subscription)
**References**		https://digitalinsights.qiagen.com	[4]	[3]	[5]	[3]

To overcome these challenges, we developed Agribio Omics Solution (AgrOmicSo), a tool that can easily analyze large-scale sequencing data and call variants by connecting the client and server. AgrOmicSo is fundamentally an interface, designed to connect a user’s local PC to a remote server so as to utilize the server’s computational power. It provides an intuitive graphical user interface (GUI) that allows users to execute complex bioinformatics pipelines without writing scripts or using the command line. This approach empowers researchers, regardless of their bioinformatics expertise, to perform comprehensive analyses, including data preprocessing, read alignment, variant calling with a choice of algorithms, and subsequent annotation and visualization. By focusing on a dedicated client-server model, AgrOmicSo aims to provide a solution that is more responsive and manageable than public web services while being more accessible and cost-effective than commercial enterprise platforms. Similar client-server architectures have been successfully applied in other scientific fields to manage data-intensive workflows; for example, Google Earth Engine in geosciences, the Rubin Science Platform in astronomy, and SWAN in high-energy physics (Table 2). These systems share a common objective, namely, decoupling user interaction from computational complexity, thereby enabling broad accessibility without compromising performance. AgrOmicSo adopts this architectural philosophy and adapts it specifically to the context of NGS data analysis.

**Table 2:** Client-server scientific workflow tools beyond NGS.

Tool Name	Domain	Client-Server/ Remote Model	Key Features	URL	References
**CARTA** (Cube Analysis and Rendering Tool for Astronomy)	Astronomy/ Radio Astronomy	Client-server (desktop client + remote server)	Interactive visualization of very large radio-astronomy image cubes (GB–TB); heavy computation and storage on remote HPC server; GPU-accelerated parallel rendering.	https://cartavis.org/	[9]
**ParaView**	Scientific Visualization (multi-domain)	Client-server (3-tier: client, data server, render server)	Interactive visualization of large simulation datasets; parallel data processing and rendering on remote HPC cluster; scalable, three-tier architecture.	https://www.paraview.org/	[10]
**Google Earth Engine**	Earth Science/ Geospatial	Cloud-based service (API-driven client)	Planetary-scale analysis of petabyte satellite/geospatial data; remote execution on Google Cloud; public data catalog with JavaScript/Python APIs.	https://earthengine.google.com/	[11]
**WebMO**	Computational Chemistry	Web-based (browser client + server)	Web GUI for quantum chemistry programs; single-server deployment (no client software needed); supports many chemistry codes and visualization in-browser.	https://www.webmo.net/	[12]
**MoleQueue**	Computational Chemistry/ HPC	Client-server (desktop GUI + local server)	Desktop system-tray app for dispatching chemistry jobs to local/remote clusters; abstracts SSH/scheduler details via JSON-RPC; integrates with common chemistry codes.	https://www.openchemistry.org/projects/molequeue/	[13]
**OPeNDAP**	Earth Science/ Climate Data	Client-server (data access protocol)	Network data access protocol for remote Earth science datasets; allows subsetting of large data over the web; many servers provide OPeNDAP endpoints for geospatial files.	https://www.opendap.org/	[14]

This paper details the architecture, implementation, features, and performance of AgrOmicSo as an interface for large-scale NGS analysis. We demonstrate its utility in streamlining complex workflows, thus making sophisticated genomic research more accessible and efficient.

## Implementation

The AgrOmicSo client application was implemented using PythonQt (version 1.8) and compiled in a Windows 11 environment via Visual Studio Code (version 1.106.3). The server-side components were developed using Python 3 on Ubuntu 22.04 and integrated widely used bioinformatics tools that were compiled or installed on the server system. Communication between the client and server was handled via custom TCP socket and FTP protocols.

## Materials and methods

### AgrOmicSo architecture

**Fig 1:**
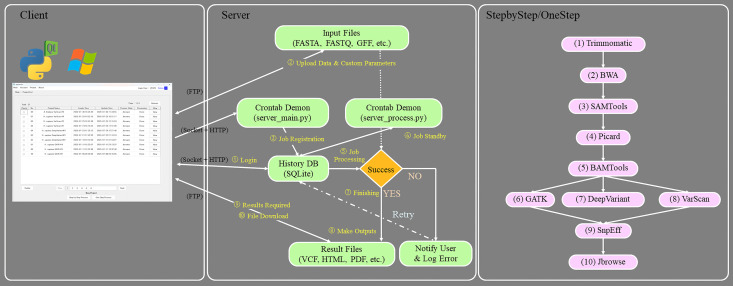
AgrOmicSo architecture and pipeline. The diagram illustrates the client-server architecture and the sequential data analysis pipeline. The client application provides the user interface (left). The server side consists of daemon processes that handle job registration and processing, communicating with the client via socket-based and FTP protocols (middle). The analysis pipeline (right) shows the sequence of bioinformatics tools executed on the server, from preprocessing to variant calling and visualization.

AgrOmicSo operates on a client-server model, wherein the client application serves as a remote-control interface for a powerful analysis engine running on a server (Fig 1). The client application, which runs on the user’s PC (Windows, macOS, or Linux), provides a GUI for project management, data input, parameter selection, and job submission (Fig 1, left panel). The server component, which is installed on a Linux-based server or HPC cluster, executes computationally intensive bioinformatics tasks. The client and server communicate via network sockets (for commands and status updates) and FTP (for data transfer) (Fig 1, middle panel). The server utilizes a local SQLite database to manage user accounts, project information, and job history, while server-side processing is orchestrated using Python scripts (server_main.py for client communication and database interaction, and server_process.py for job execution). This architecture allows multiple users to securely and simultaneously access the server’s computational resources from their individual client applications. However, the server for AgrOmicSo does not depend on HPC queuing systems such as Slurm or PBS; it directly manages and runs jobs without using a queuing system.

The client initiates control requests using TCP socket communication (default port 7000), and file transfers (such as HTML report and VCF output) are handled via FTP (default port 21), as defined in the system configuration (server.cfg). While this architecture facilitates fast and straightforward communication, the current implementation protocol does not include end-to-end encryption. Basic security measures are implemented, including user-level authentication and task segregation on the server. The server monitors task execution via internal logging and controls job access based on user identity. However, as FTP and socket communication are currently unencrypted, these mechanisms are better suited for securing internal networks (e.g., within institutional HPC infrastructure). To enhance security for broader deployment, future updates will incorporate encrypted communication protocols—specifically, by replacing FTP with SFTP and wrapping socket-based messaging with SSL/TLS. These improvements will ensure secure data transmission and strengthen compliance with common institutional cybersecurity requirements.

### Bioinformatics pipeline

AgrOmicSo integrates a series of well-established bioinformatics tools into a cohesive pipeline, designed to follow best practices for variant discovery. The tools included in AgrOmicSo were carefully chosen according to the pipeline used by the National Agricultural Biotechnology Information Center (NABIC, Republic of Korea; Fig 1).

1. **Data Preprocessing and Quality Control:** The pipeline begins with an assessment of raw sequencing data quality using FastQC (https://www.bioinformatics.babraham.ac.uk/projects/fastqc/). Based on this assessment, users can proceed with data cleaning using Trimmomatic [15]. This step is critical for removing adapter sequences, trimming low-quality bases from the ends of reads, and filtering out reads that are too short after trimming, thereby reducing noise and improving the accuracy of downstream alignment.
2. **Read Mapping:** The cleaned, high-quality reads are aligned to a user-provided reference genome (in FASTA format) using the BWA-MEM algorithm from the Burrows-Wheeler Aligner package [9]. This aligner is optimized for long reads and split-read alignments, making it robust for variant detection.
3. **Post-Alignment Processing:** The initial alignment output (SAM format) is converted to the compressed binary format (BAM), sorted by coordinate, and indexed using SAMtools [10]. This processing is essential for efficient data handling and visualization. Following this, Picard tools (http://broadinstitute.github.io/picard/) are used for further refinement. The FixMateInformation command ensures that mate-pair information is consistent and correct, while MarkDuplicates identifies and flags PCR duplicates that can otherwise lead to false positive variant calls. Statistics on the alignment can be calculated using BamTools [11].
4. **Variant Calling:** AgrOmicSo empowers users by offering a selection of three distinct and widely used variant calling algorithms. This flexibility allows researchers to choose the most suitable tool for their data and experimental goals.
  - **GATK (HaplotypeCaller) [5]:** A community-standard tool that performs local de novo assembly of haplotypes to accurately call SNPs and INDELs.
  - **DeepVariant [6]:** A deep learning-based variant caller from Google that reframes variant calling as an image classification problem, known for its high accuracy on various sequencing platforms.
  - **VarScan 2 [7]:** A robust, heuristic tool that is particularly effective for detecting variants in diverse datasets, including those with low coverage or pooled samples.
5. **Variant Annotation and Visualization:** After variant calling, the resulting VCF file is annotated using SnpEff [12] to predict the functional effects of the detected variants (e.g., missense, nonsense, frameshift). Finally, the results, including the aligned reads (BAM) and annotated variants (VCF), can be loaded into an integrated JBrowse [13] instance for interactive visualization and manual inspection.

During pipeline execution, the server continuously tracks each processing step via internal logging. If a failure occurs at any stage (e.g., mapping, post-alignment processing, or variant calling), the job is immediately halted and the error is recorded. The client interface reports the failure to the user, thus allowing the pipeline to be resumed from the failed step without restarting the entire analysis (Table 3).

**Table 3:** Tools included in the AgrOmicSo software.

Step	Tool	Version	Reference
QC	FastQC	0.11.5	(https://www.bioinformatics.babraham.ac.uk/projects/fastqc/)
	TrimmOmatic	0.36	[9]
Alignment	BWA	0.7.16a	[10]
Post-processing	Samtools	0.1.18	[11]
	Picard	2.9.4	(http://broadinstitute.github.io/picard/)
	BamTools	2.4.2	[12]
	GATK (IndelRealigner)	3.7.0	[6]
Variant calling	GATK (HaplotypeCaller)	3.7.0	[6]
	GATK (UnifiedGenotyper)	3.7.0	[6]
	DeepVariant	0.5.1	[7]
	VarScan 2	2.3.9	[8]
Variant annotation	SnpEff	4.3q	[13]
Visualization	Jbrowse	1.12.3	[15]

The tools are listed in the order of their use in the pipeline.

### Modes of operation

- **One-Step Process Mode:** This mode allows users to run the entire pipeline from raw reads to annotated variants with a single submission, using default or pre-set optimized parameters for each tool. This is ideal for batch processing multiple samples or for users who prefer a fully automated workflow without manual intervention.
- **Step-by-Step Process Mode:** This interactive mode provides greater control, allowing users to execute each step of the pipeline individually. Users can review intermediate results (e.g., FastQC reports, alignment statistics), adjust parameters for subsequent steps, and make informed decisions throughout the analysis. This mode is suitable for expert users, troubleshooting, or custom, exploratory data analysis.

These complementary modes are designed to accommodate users with varying levels of bioinformatics expertise and analysis goals. By providing both streamlined automation and customizable control, AgrOmicSo enables users to choose the most appropriate workflow strategy for their specific data size, research question, and technical comfort level. The one-step process mode is well-suited for large-scale analyses of multiple samples processed using consistent parameters, while the step-by-step process mode is useful in the early stages of data exploration when fine-tuned settings and preliminary quality assessments are required. Together, these modes allow AgrOmicSo to support high-throughput genomic analysis in an efficient and precise manner.

### Client program installation and server configuration

The AgrOmicSo client program is implemented in Python with PythonQt for the GUI and can be run on Windows, macOS, and Linux. The client package includes the necessary executable image files, the JBrowse folder, and a server.cfg configuration file. This file stores the server IP address, port number, FTP credentials, and path to the local JBrowse executable.

The server-side setup requires a Linux environment. It involves creating a user account for AgrOmicSo, registering the server_main.py and server_process.py scripts as server daemons, setting up an SQLite database for user and job management, and ensuring that the necessary communication ports are open. All third-party bioinformatics tools used in the pipeline must be installed and accessible on the server’s system path. Currently, third-party tools must be manually updated via the Conda environment. Please see the Discussion for limitations and future plans.

### Benchmark analyses

To evaluate the performance and reproducibility of variant calling tools integrated into AgrOmicSo, benchmarking experiments were conducted using whole-genome sequencing data from three different species: *Arabidopsis thaliana* (SRR21871726, SRR21871728, SRR21871730), *Xanthomonas oryzae pv. oryzicola GX01* (11139_1, 11139_2, 11139_3), and *Homo sapiens* (SRR18574455, SRR18574456, SRR18574457). For each species, three biological replicates were used.

Variant calling was performed using three widely used tools—GATK4, DeepVariant, and VarScan2—resulting in nine experimental conditions (3 species × 3 tools). Each condition was executed independently using AgrOmicSo and Galaxy, producing a total of 18 test cases. The variant calling pipelines were configured on both platforms to ensure consistency in preprocessing, alignment, and variant filtering steps.

All tests were conducted on a local server running Ubuntu 20.04.6 LTS with an Intel® Xeon® CPU Gold 6240R CPU @ 2.40 GHz and 1,024 GB of RAM. For each run, we recorded the total execution time and number of detected variants, including SNPs and INDELs. To assess reproducibility and tool-specific variability, each variant calling workflow was repeated three times per condition.

### Statistical analyses

Statistical analyses were performed to evaluate the differences in variant calling performance and quantify the sources of variability between tools and platforms. All statistical analyses were conducted using Python (SciPy, NumPy, and Statsmodels). To assess reproducibility, the coefficient of variation (CV = standard deviation/mean) of SNP counts was calculated across three replicates of the *A. thaliana* dataset for each variant caller on both AgrOmicSo and Galaxy platforms. CVs were visualized on a logarithmic scale to highlight differences in variability among tools. To determine whether observed differences in SNP counts were attributable to the execution platform or variant calling algorithm, variance decomposition was performed using two-way ANOVA. Effect sizes (η²) were calculated to quantify the relative contribution of platform, tool, and their interaction to the total variance. When variance was present, Welch’s t-test was applied for direct comparisons between the same tool executed on different platforms. In cases where variance was minimal or zero across replicates, permutation tests were used to determine robust p-values independent of variance assumptions. Effect sizes were reported as mean differences with bootstrap confidence intervals. When comparing multiple variant callers under the same platform and dataset, one-way ANOVA was used to assess differences in SNP counts among tools. Boxplots and bar plots were generated to visualize group variability and reproducibility. These analyses enabled rigorous benchmarking of tool performance, reproducibility, and platform consistency.

## Results

### An accessible interface for server-based genomic analysis

AgrOmicSo successfully implements a client-server model to provide an accessible interface for complex NGS data analysis. Users can manage projects, transfer large sequencing files to a server, define analysis parameters, and launch jobs through a simple GUI without direct command-line interaction. The client application provides real-time feedback on the status of each processing step, from “Ready” and “Running” to “Done.” This architecture effectively decouples the user’s workspace from the computational workload, allowing for stable, long-running analyses of large datasets on a remote, powerful machine. The design philosophy prioritizes ease of use, enabling genomics researchers who wish to analyze population-level data to do so without needing to write or manage complex scripts.

### Performance comparison of integrated variant callers

The benchmark analysis using *X. oryzae pv. oryzicola GX01* data revealed significant performance differences among the three integrated variant callers, providing users with a clear choice based on their experimental priorities.

In terms of execution time, VarScan was the fastest, completing the variant calling step in approximately 14 minutes (Table 4). GATK was significantly slower, requiring over 17 minutes, while DeepVariant’s execution time was intermediate at around 16 minutes. However, in variant calling using *A. thaliana* and *H. sapiens* data, VarScan was the fastest, followed by GATK and then DeepVariant.

**Table 4:** Comparison of execution time for variant calling algorithms.

*Xanthomonas oryzae pathovar oryzicola GX01*				
Algorithms	Times (hh:mm:ss)			
	1^st^	2nd	3^rd^	Average
DeepVariant	00:17:15	00:16:47	00:17:18	00:17:07
GATK	00:16:11	00:16:00	00:16:43	00:16:18
VarScan	00:14:24	00:13:56	00:14:12	00:14:11
***Arabidopsis Thaliana***				
	Start	End	Time	
GATK	14:14:32	14:23:06	00:08:34	
DeepVariant	18:45:01	20:02:59	01:17:58	
VarScan	17:03:36	17:08:46	00:05:10	
***Homo sapiens***				
GATK	13:43:13	16:24:37	02:41:24	
DeepVariant	09:51:18	12:11:19	02:20:01	
VarScan	19:56:58	21:30:06	01:33:08	

Regarding variant detection, DeepVariant identified the highest number of total variants (220,860), followed by GATK (182,403), and VarScan (167,509) (Table 5). This trend was consistent for both SNPs and INDELs, although VarScan identified a notably lower number of INDELs compared to GATK and DeepVariant. DeepVariant detected the largest number of variants in both *A. thaliana* and *H. sapiens*, followed by GATK and VarScan. In particular, DeepVariant took 26:20:01 in H. sapiens, but called significantly more variants (1,330) than GATK or VarScan.

**Table 5:** Comparison of variant call results.

*Xanthomonas oryzae pathovar oryzicola GX01*					
Algorithms	Variants				
	Total	Unique	Intersection		
DeepVariant+GATK+VarScan			1,58,152		
			DeepVariant	GATK	VarScan
DeepVariant	2,20,860	44,328		17,014	1,366
GATK	1,82,403	3,095	17,014		4,142
VarScan	1,67,509	3,849	1,366	4,142	
***Arabidopsis Thaliana***					
Algorithms	Variants				
	Total	Unique	Intersection		
DeepVariant+GATK+VarScan			1,58,165		
			DeepVariant	GATK	VarScan
DeepVariant	2,20,860	44,286		17,056	1,353
GATK	1,82,460	3,100	17,056		4,139
VarScan	1,67,509	3,852	1,353	4,139	
***Homo sapiens***					
Algorithms	Variants				
	Total	Unique	Intersection		
DeepVariant+GATK+VarScan			7		
			DeepVariant	GATK	VarScan
DeepVariant	1,330	1,310		20	0
GATK	20	0	20		0
VarScan	9	2	0	0	

**Fig 2:**
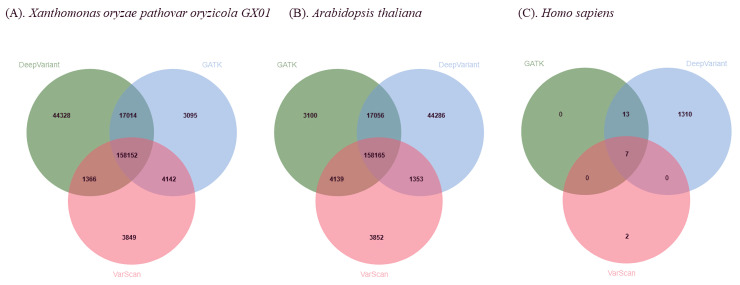
Comparison of variants called by GATK, DeepVariant, and VarScan in *Xanthomonas oryzae pv. oryzicola GX01*, *Arabidopsis thaliana*, and *Homo sapiens.* **(A)** Venn diagram illustrates the overlap and unique variants identified by each of the three calling algorithms in *X. oryzae pv. oryzicola GX01*. **(B)** Venn diagram illustrates the overlap and unique variants identified by each of the three calling algorithms in *A. thaliana*. **(C)** Venn diagram illustrates the overlap and unique variants identified by each of the three calling algorithms in *H. sapiens*.

A Venn diagram illustrates the overlap between the variant call sets (Fig 2). A substantial number of variants (158,152 in *X. oryzae pathovar oryzicola GX01*, 158,165 in *A. thaliana*, and 7 in *H. sapiens*) were commonly identified by all three algorithms, representing a high confidence set. However, each algorithm also identified a unique set of variants. Notably, DeepVariant called 44,328 unique variants, 44,286 unique variants and 1,310 unique variants from *X. oryzae pv. oryzicola GX01*, *A. thaliana*, and *H. sapiens*, respectively, which were not found by the other two algorithms, highlighting their high sensitivity. These results demonstrate that AgrOmicSo provides users with critical flexibility, allowing them to choose an algorithm that best fits their research priorities—be it speed (VarScan), high sensitivity (DeepVariant), or the use of a community-standard tool (GATK).

### Reproducibility of SNP detection across replicates

**Fig 3:**
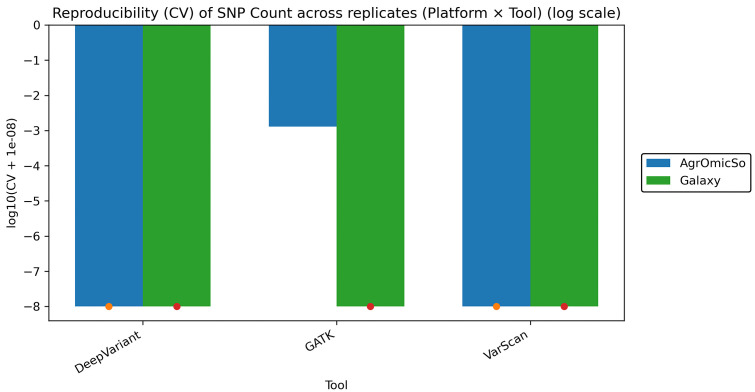
Reproducibility of SNP detection across variant callers measured by coefficient of variation (CV) across replicates (log scale). The CV of SNP counts across three replicates was calculated for each variant caller on both platforms. Values are shown on a log scale to emphasize differences in reproducibility. DeepVariant and GATK exhibited extremely low variability across replicates, whereas VarScan showed substantially higher variability in SNP detection on both platforms.

To assess the stability of variant detection, we evaluated the reproducibility of SNP counts across three replicates of the *A. thaliana* dataset for each variant caller on both AgrOmicSo and Galaxy platforms. The CV was calculated and visualized on a log scale (Fig 3).

DeepVariant and GATK exhibited extremely low variability across replicates, with CVs approaching zero, indicating highly stable and reproducible SNP detection in the *A. thaliana* dataset. In contrast, VarScan showed markedly higher variability, with CVs being several orders of magnitude larger. Importantly, this pattern was consistent across both AgrOmicSo and Galaxy, demonstrating that reproducibility is primarily determined by the variant calling algorithm rather than by the execution platform. This analysis provides a quantitative measure of variability and highlights reproducibility as a critical factor when selecting a variant calling tool.

### Source of variability in SNP counts: Two-way ANOVA

**Fig 4:**
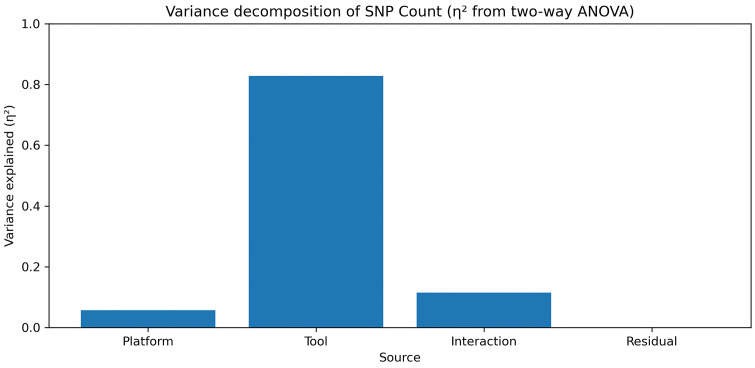
Variance decomposition of SNP count using two-way ANOVA (η² effect size). Two-way ANOVA was used to decompose the total variance of SNP counts into contributions from platform, variant caller (tool), and their interaction. The effect size (η²) indicates that the vast majority of variance is explained by the variant calling tool, while the contribution from the execution platform is comparatively small.

To determine whether differences in SNP counts were driven by the execution platform or the variant calling tool, we performed variance decomposition using two-way ANOVA and calculated effect sizes (η²) (Fig 4).

More than 80% of the total variance in SNP counts was attributable to the variant calling tool, while the contribution from the platform (AgrOmicSo vs Galaxy) was minimal. The interaction effect between the platform and tool was also small compared to the tool effect. These results confirm that observed differences in SNP counts are predominantly due to intrinsic differences between variant calling algorithms rather than differences between computational environments.

### Summary of benchmark analyses findings

Together, these results demonstrate that AgrOmicSo provides users not only with flexibility in choosing variant callers based on speed and sensitivity, but also insight into the reproducibility characteristics of each algorithm. While VarScan offers the shortest execution time, DeepVariant and GATK provide more stable and reproducible SNP detection across replicates. Variance decomposition analysis further showed that these differences are inherent to the algorithms themselves and not influenced by the execution platform. This integrated evaluation of speed, sensitivity, and reproducibility enables informed selection of variant calling tools within the AgrOmicSo environment.

## Discussion

We present AgrOmicSo as a comprehensive tool for NGS data analysis, offering robust features and a user-friendly environment. Its primary innovation lies in its dedicated client-server architecture, which is designed specifically to provide a simple, graphical interface to powerful server-side computational resources. This model directly addresses a major bottleneck in modern genomics: the difficulty many researchers face when trying to analyze large datasets on command-line-based HPC systems. By separating the user interface (client) from the computational engine (server), AgrOmicSo empowers researchers to manage and execute large-scale analyses without needing advanced bioinformatics skills.

A key advantage of AgrOmicSo is the integration of multiple variant calling algorithms (GATK, DeepVariant, and VarScan). As demonstrated by our benchmark analyses, different variant callers exhibit trade-offs between speed, sensitivity, and the types of variants detected. In addition to these trade-offs, our variability analyses revealed that reproducibility depends strongly on the chosen variant caller. SNP counts produced by DeepVariant and GATK were highly consistent across replicates, whereas VarScan showed substantially higher variability. Variance decomposition further supported this observation, indicating that most of the variability in SNP counts was explained by the variant calling tool itself, with only a minor contribution from the execution platform (AgrOmicSo vs Galaxy). These findings suggest that users should consider reproducibility metrics along with runtime or sensitivity, when selecting a variant calling algorithm for downstream analyses. VarScan offers the fastest performance, making it suitable for rapid, preliminary analyses. DeepVariant provides the highest sensitivity, which is crucial for studies aiming to discover rare or novel variants. GATK remains a widely used standard, and its inclusion ensures comparability with a large body of existing literature. By providing these options within a single interface, AgrOmicSo allows researchers to select the most appropriate tool for their specific needs, or even to combine the results from multiple callers to generate high-confidence variant sets. If one wishes to perform variant calling quickly, we recommend using the VarScan algorithm, but it must be noted that the number of variants will be smaller than other algorithms. If one wishes to call many variants even if it takes time, we recommend using the GATK or DeepVariant algorithm.

Compared to other platforms (Table 1), AgrOmicSo occupies a unique niche. Unlike monolithic, often costly commercial platforms such as the CLC Genomics Server, AgrOmicSo is open-source and provides users with full control over their own server environment. Compared to highly flexible but complex web platforms like Galaxy, AgrOmicSo offers a more streamlined and dedicated user experience focused on a core, high-demand analysis pipeline. This makes it an ideal solution for individual labs or institutions that wish to establish a centralized, easy-to-use analysis service on their own hardware.

Furthermore, the need for computationally and energetically efficient software is becoming increasingly critical. As recent events have shown, extreme weather can impact the operation of even large-scale data centers, making “green computing” not just an environmental goal but a practical necessity. While this study did not repeat the power consumption benchmarks from previous works, the principle remains vital. By enabling analysis on efficient, centralized servers and providing choices between algorithms with different performance profiles, AgrOmicSo provides a framework for more sustainable computational research compared to numerous, less efficient local machines.

While AgrOmicSo offers substantial improvements in usability and accessibility, it is important to acknowledge current limitations. One limitation of this study is that the statistical variability analysis was performed using only the *A. thaliana* dataset. This choice was intentional, as AgrOmicSo was originally designed to support agricultural and plant genomics research, and *A. thaliana* serves as a well-established model organism in plant genomics with extensively validated reference data. We therefore selected this dataset as a representative case for evaluating reproducibility and variance sources in variant calling. Future work will extend this statistical analysis to additional organisms and sequencing datasets to further generalize these findings. Another limitation of AgrOmicSo is that it currently supports only Illumina format among NGS data. The server-side installation, while documented, requires some system administration knowledge. In the future, we plan to expand its features to support data in various formats such as IonTorrent, Oxford Nanopore Technology, and PacBio. We also recognize that installing the server-side program of AgrOmicSo can still be challenging owing to the numerous external software dependencies, even with the requirements.txt and environment.yml files provided. To address this, we plan to simplify the server deployment process by providing a Docker image or an automated installation script in a future version release. This will allow users to deploy AgrOmicSo and all required dependencies in a single step, significantly lowering the barrier to adoption and improving accessibility for users without experience in advanced system administration. Moreover, the server of AgrOmicSo does not rely on HPC queuing systems such as Slurm or PBS, but it directly manages and runs jobs. This is an important limitation of AgrOmicSo as a client-server pipeline. We intend to develop AgrOmicSo to enable the use of HPC queuing systems in the next version. Additionally, AgrOmicSo is currently configured to accommodate specific versions of bioinformatics tools and libraries, but users can replace these with newer versions by modifying the environment settings if needed. Since AgrOmicSo manages package dependencies through a Conda environment, users can update individual tools by editing the provided environment.yml file or using Conda commands. However, a one-step update feature has not yet been implemented. We plan to add a package update feature in the future to improve user convenience. Finally, we will incorporate additional analysis modules that support other common NGS applications, such as RNA-Seq or metagenomics.

In conclusion, AgrOmicSo represents a valuable tool for the genomics research community. Its combination of a user-friendly interface, robust client-server architecture, and flexible pipeline with options for calling multiple variants, positions it to facilitate a wide range of NGS-based studies. By simplifying complex bioinformatics workflows, AgrOmicSo aims to accelerate the pace of discovery in genomics.

## Supporting information

S1 File Supplementary. (DOCX)
