# Supplementary material for: AgrOmicSo: A client-server interface for accessible large-scale analysis of next-generation sequencing data
Source: PLoS One. 2026 Jun 1;21(6):e0348571. doi: 10.1371/journal.pone.0348571 (PMC13225662; doi:10.1371/journal.pone.0348571)
Supplement: S1 File — (DOCX) [file pone.0348571.s001.docx]

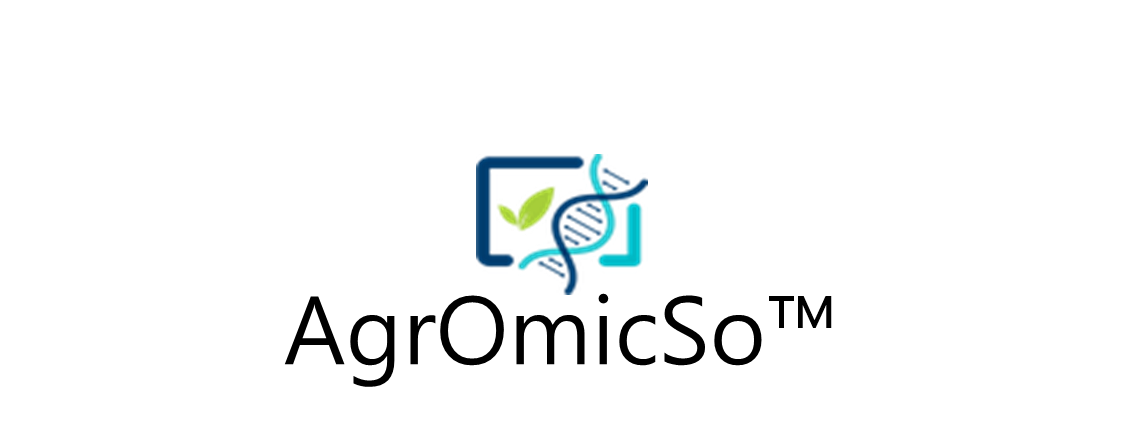


AgrOmicSo(Agri-bio Omics Solution)

User Manual

**Slide 1**

#
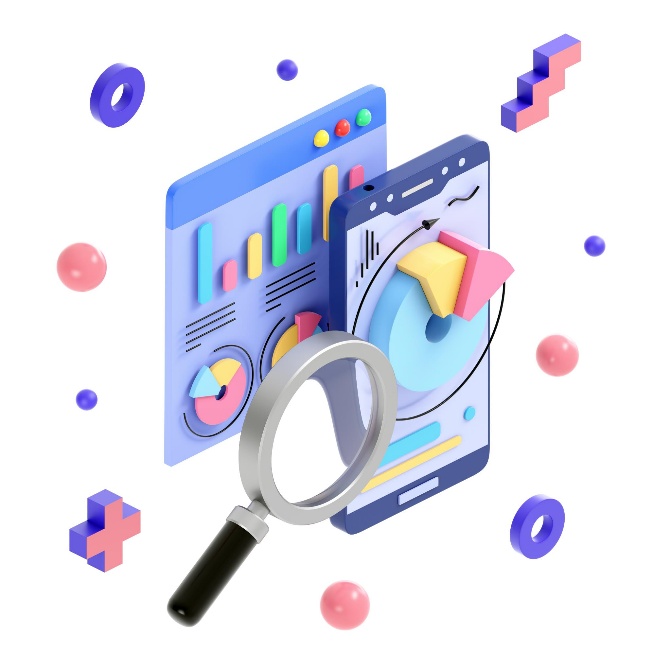
The Challenges of Analyzing NGS Data

The Importance of Accuracy

- The accuracy of NGS data analysis is highly dependent on data quality, which significantly impacts research outcomes.

The Impact of Low Quality

- Low-quality data can lead to incorrect variant analysis, which can lead to incorrect conclusions.

The Importance of Follow-up Analysis

- Ensuring the accuracy of variant analysis is essential to improving the accuracy of follow-up analyses.

**Slide 2**

# Command Line NGS Data Analysis


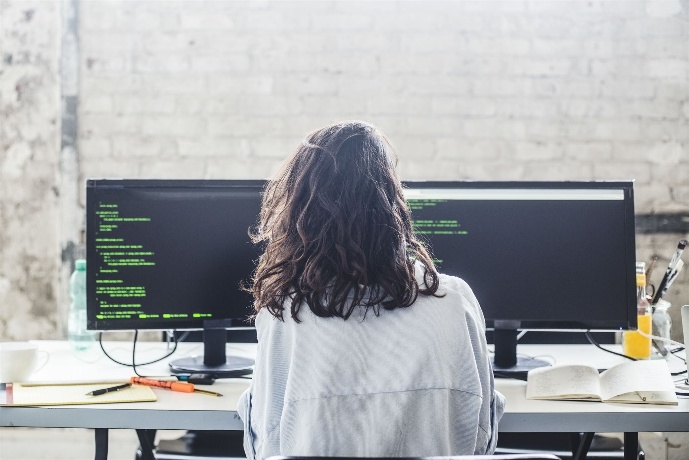
The Need for Expertise

- Understanding advanced bioinformatics requires a significant level of expertise.

Barriers and Challenges

- Command-based mutation calling poses a significant barrier for non-experts. → Limited Accessibility

The Need for Education

- Education and training are needed to enable beginners and non-experts to enter the field of bioinformatics

**Slide 3**

#
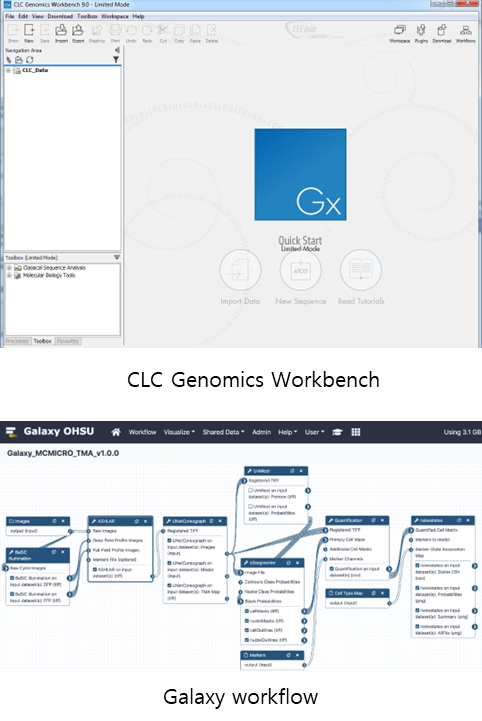
Workflow-based NGS Data Analysis

Support for a variety of bioinformatics analysis tools

- Workflow-based platforms offer a variety of bioinformatics analysis tools.
- Empower researchers to easily analyze data.

Complex initial setup

- Initial setup of workflow-based platforms can be complex for users to understand.
- Beginners may need assistance.

Complexity of advanced features

- Using advanced features requires additional learning.

**Slide 4**

# Introduction to AgrOmicSo™


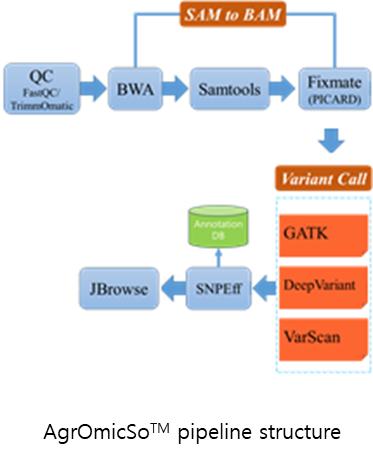
AgrOmicSo is a versatile software application designed for automated analysis of next-generation sequencing (NGS) data. It integrates various functions, such as data preprocessing, read mapping, and variant analysis, to enable efficient and accurate genome analysis.

AgrOmicso's Structure and Features

1. Data Preprocessing:

Removing noise and errors from sequencing data (Trimming function)

☞ Improving data quality

1. Read Mapping:
   1. Mapping NGS sequencing reads to reference genomes or registered genome sequences
   2. Error minimization steps (read filtering and deduplication, etc.)

**Slide 5**

# Introduction to AgrOmicSo™

AgrOmicSo is a versatile software application designed for automated analysis of next-generation sequencing (NGS) data. It integrates various functions, such as data preprocessing, read mapping, and variant analysis, to enable efficient and accurate genome analysis.


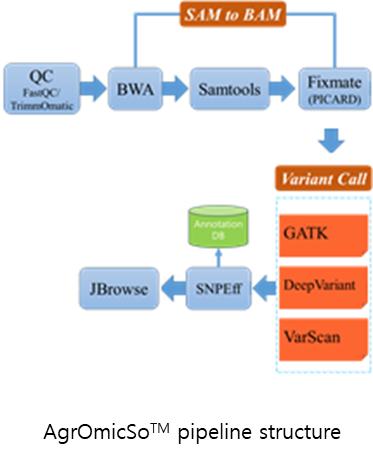


AgrOmicso's Structure and Features

1. Mutation Analysis:

Identifies genetic variants

☞ Supports exploration of genomic diversity and disease mechanism research

1. Genome Browser Integration:

Visually visualizes analysis results

☞ Enables intuitive understanding and rapid data retrieval

1. User-Friendly Interface:

Streamlined interface, automated processes

☞ Accessible even to individuals without bioinformatics training

**Slide 6**

#
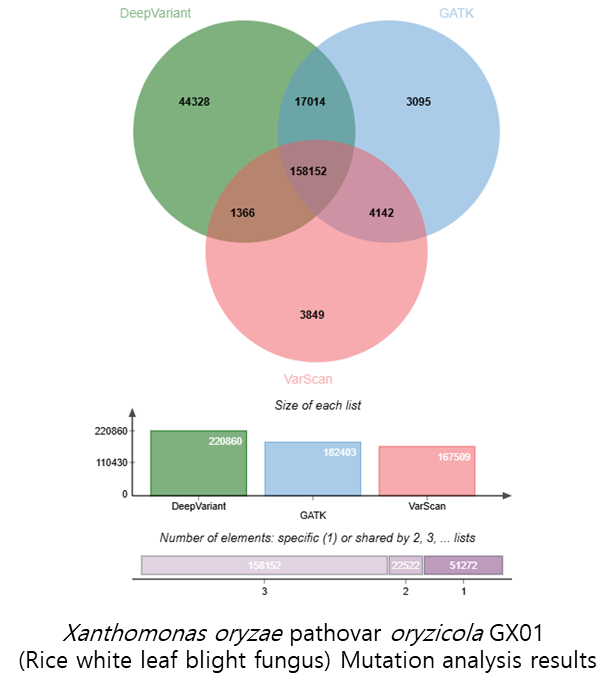
Introduction to AgrOmicSo™

AgrOmicSo's Unique Advantages

**> Client-Server Support (Supercomputer): Next Version**

Most existing NGS analysis tools are PC-only or require server installation

- There are no tools capable of analyzing large-scale sequencing data through client-server communication.
- AgrOmicSo analyzes, visualizes, and manages job history for large-scale sequencing data through client-server communication.
- Support for distributed processing through the server's job manager improves the speed of large-scale sequencing data analysis.

**Slide 7**

#
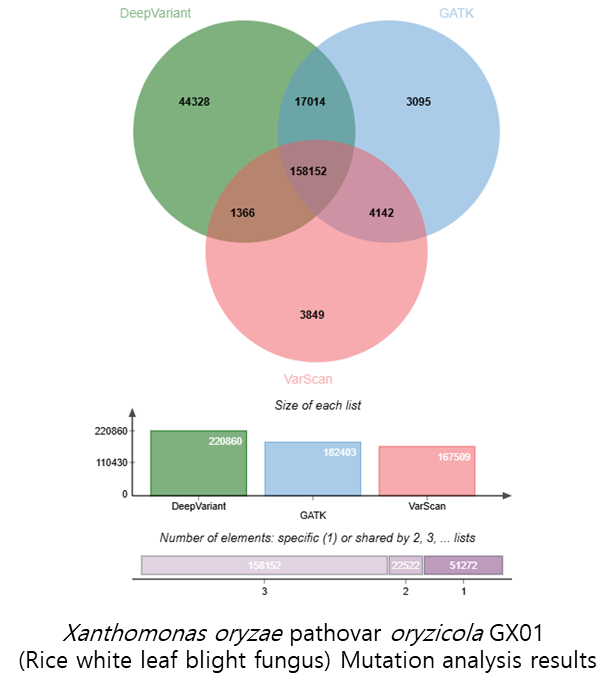
Introduction to AgrOmicSo™

AgrOmicSo's Unique Advantages

**> Client-Server Support (Supercomputer): Next Version**

**> Easy-to-use interface**

- No complex setup required, easy-to-use interface enables quick and easy analysis of large amounts of sequencing data
- Detailed options available for advanced users
- One-step process mode for faster analysis

**Slide 8**

#
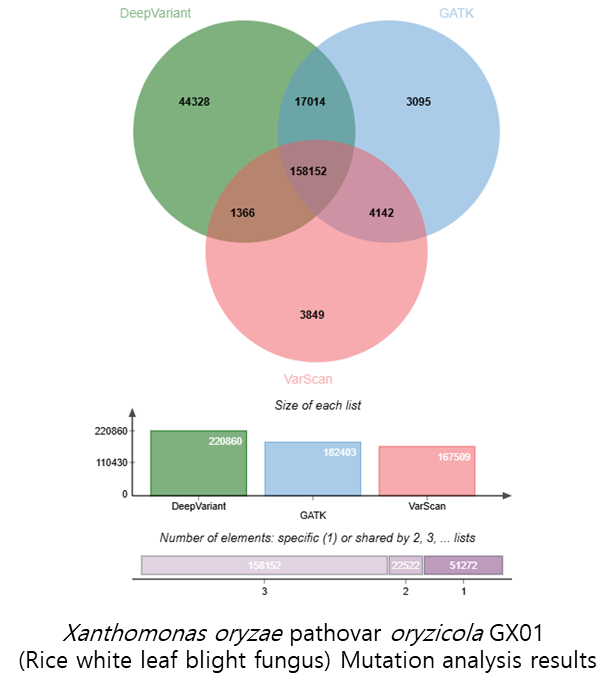
Introduction to AgrOmicSo™

AgrOmicSo's Unique Advantages

**> Client-Server Support (Supercomputer): Next Version**

**> Easy-to-use interface**

**> Supports various mutation analysis algorithms**

- Available to use various mutation analysis algorithms

☞ You can analyze mutation information not found when using each algorithm alone

- Available to analyze mutation information from one or more genome sequencing data

☞ Population-level genotyping possible

☞ Can be used for breeding or disease-causing gene discovery

**Slide 9**

# AgrOmicSo™ How to Use - 1. Connection

- Server account required.
- Connect to the specified server address and port.


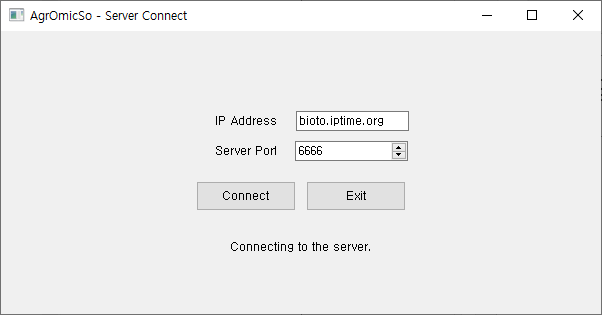

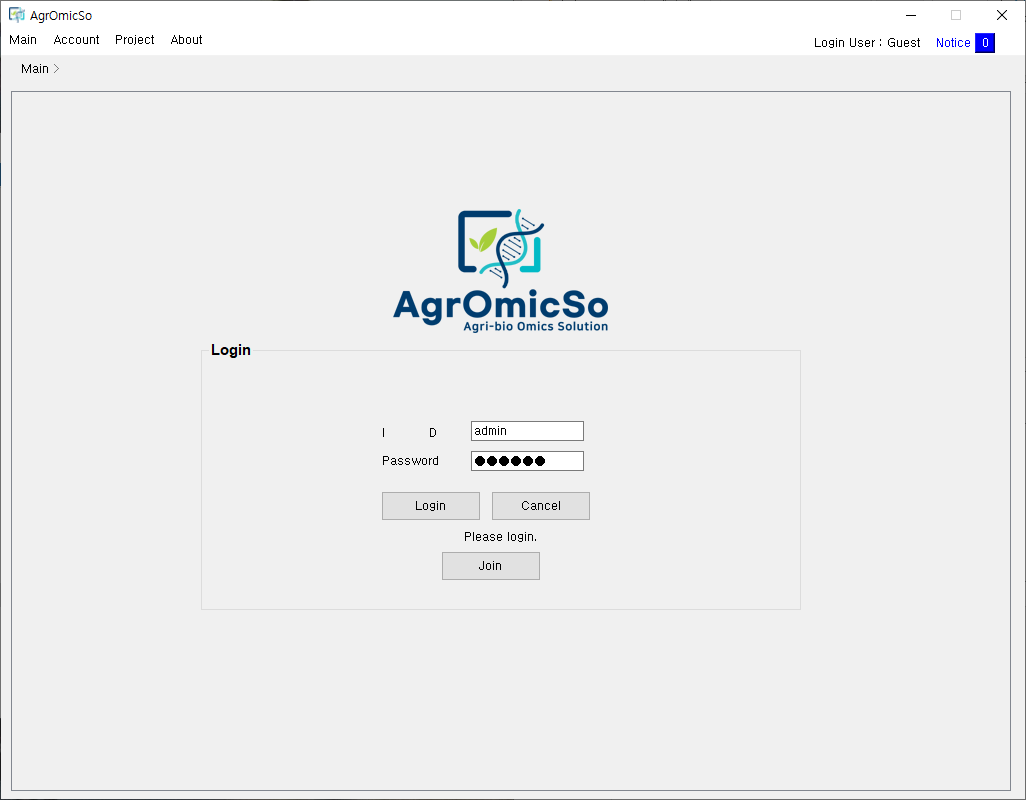


**Slide 10**

# AgrOmicSo™ How to Use - 2. Proj ect List

- Create a project or check project details.
- New project creation supports two modes: Step-by-Step and One-Step.

**
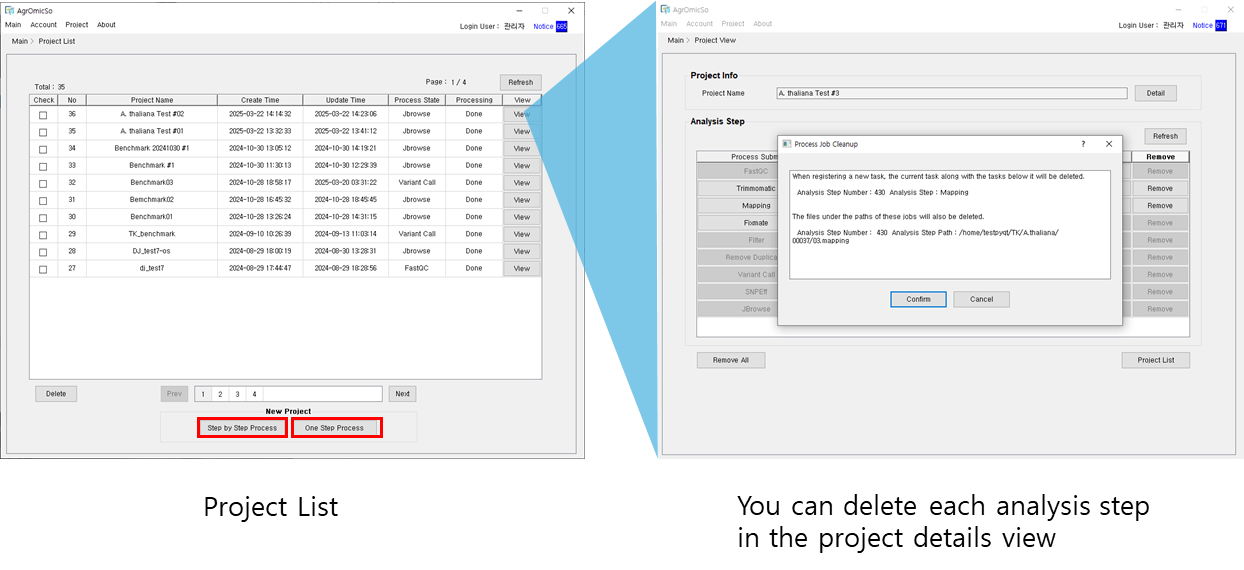
**

**Slide 11**

# AgrOmicSo™ How to Use - 3. New Project (Step by Step Process)

**
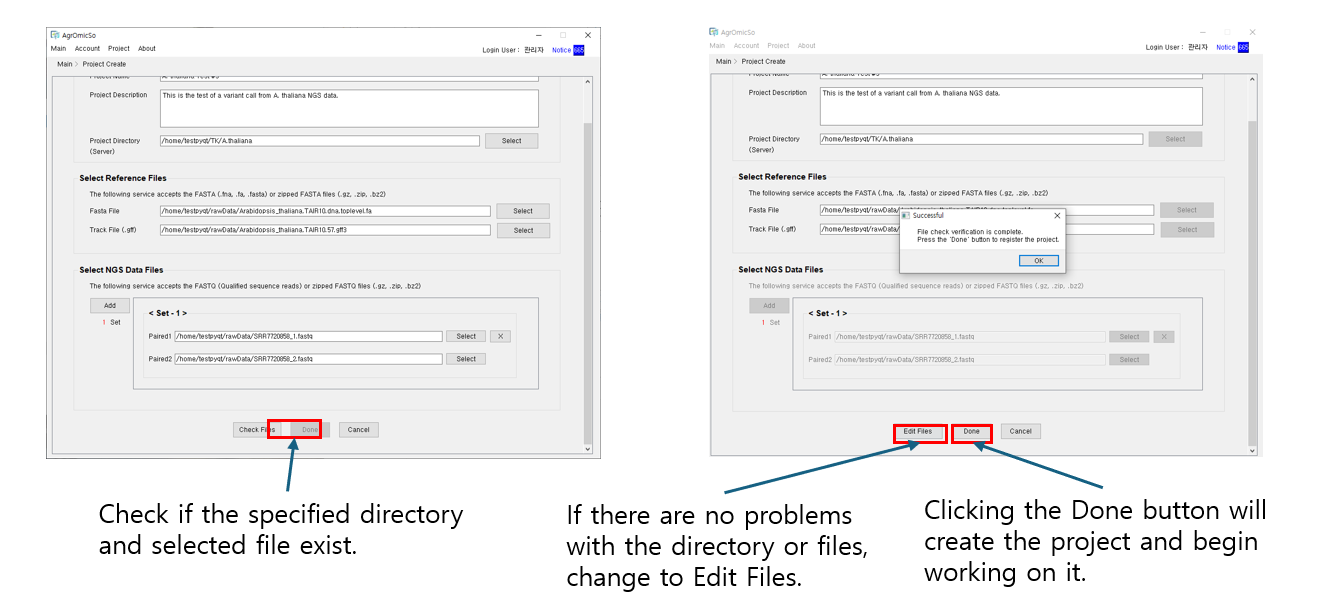
**

- Select Reference File
- Select NGS Data
- Specify a Project Name
- Specify a Project Directory

**Slide 12**

# AgrOmicSo™ How to Use - 4. Project List (Monitoring)

- Check the progress of each project.
- Click the View button to view detailed step-by-step status and results.

**
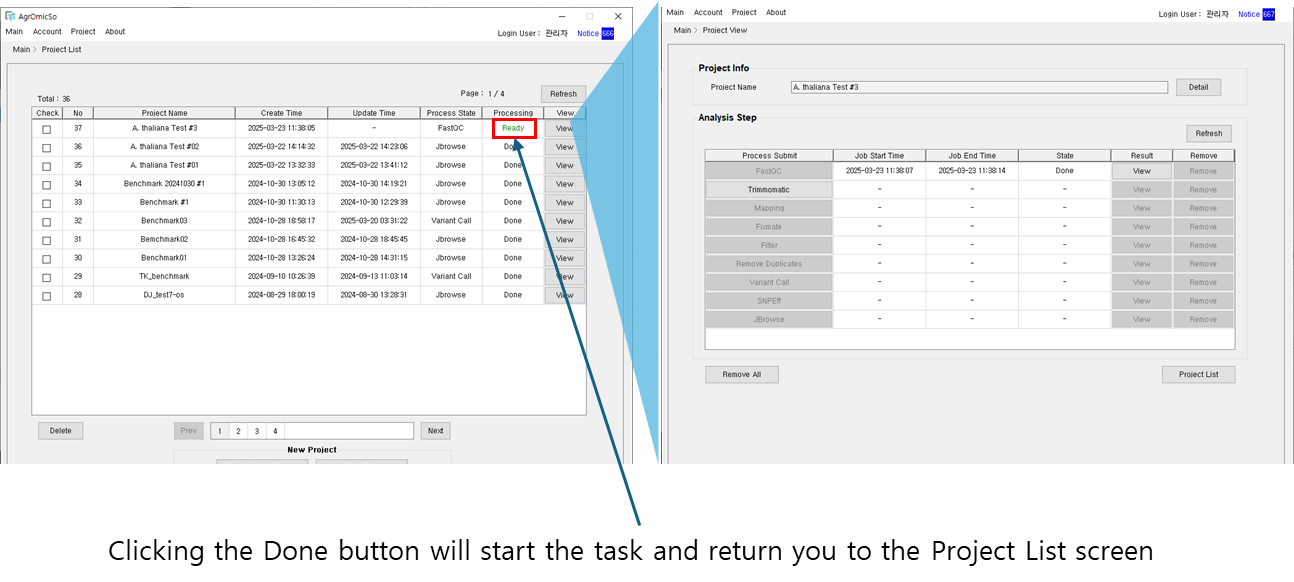
**

**Slide 13**

# AgrOmicSo™ How to Use - 5. Check FastQC Results

- Check the sequencing quality of NGS raw data.
- QC Report button: Check the results file in your PC's default web browser.

**
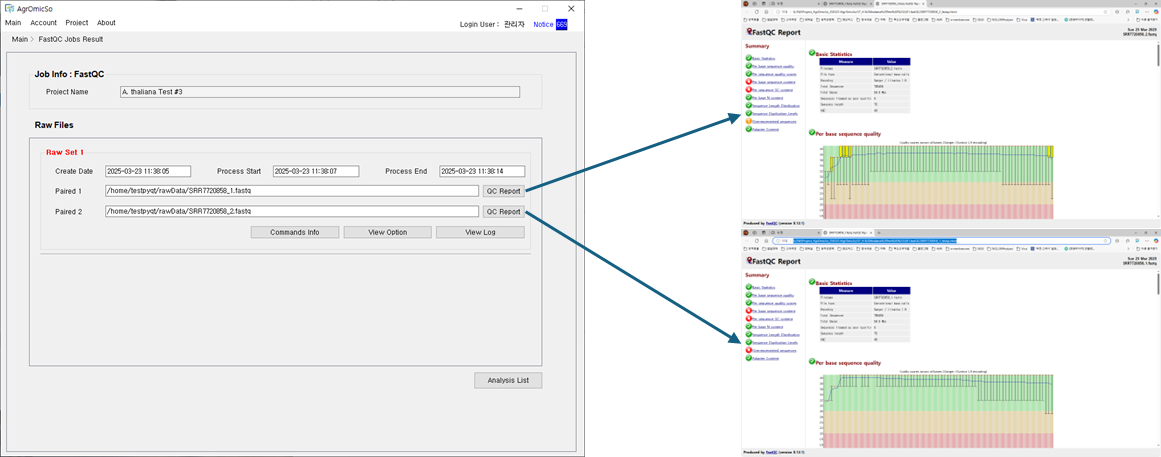
**

**Slide 14**

# AgrOmicSo™ How to Use - 6. Run Trimmomatic

- After verifying the quality of NGS raw data with FastQC, this step removes low-quality sequences.
- The next step cannot be executed until the previous step is completed.

**
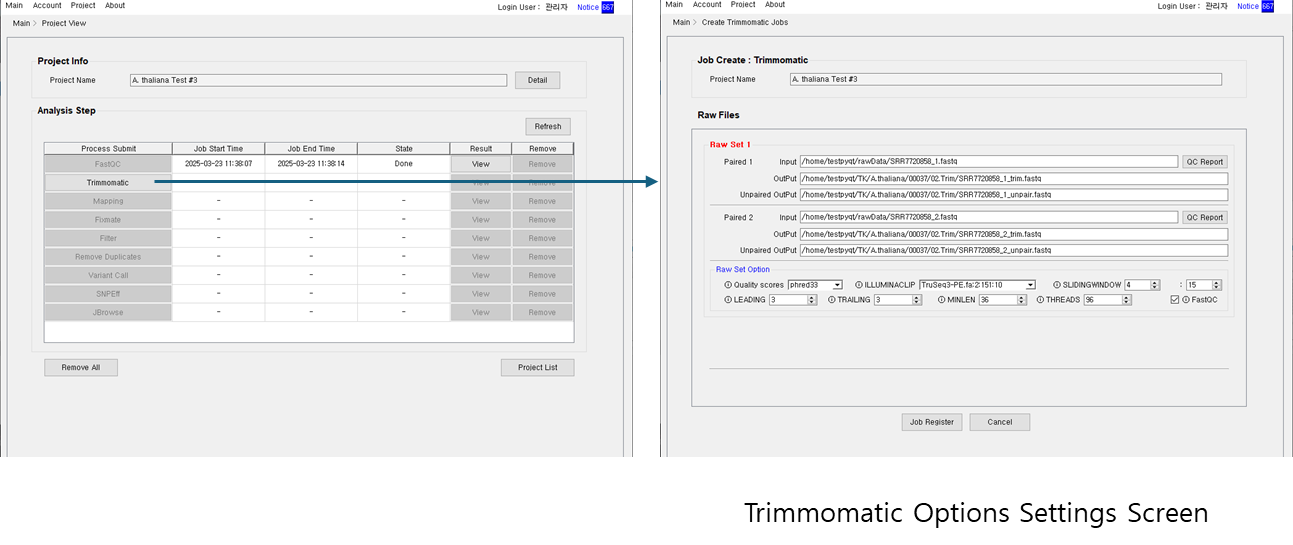
**

**Slide 15**

# AgrOmicSo™ How to Use - 6. Check Trimmomatic Results

- Confirm the results of removing low-quality sequences from NGS raw data.

**
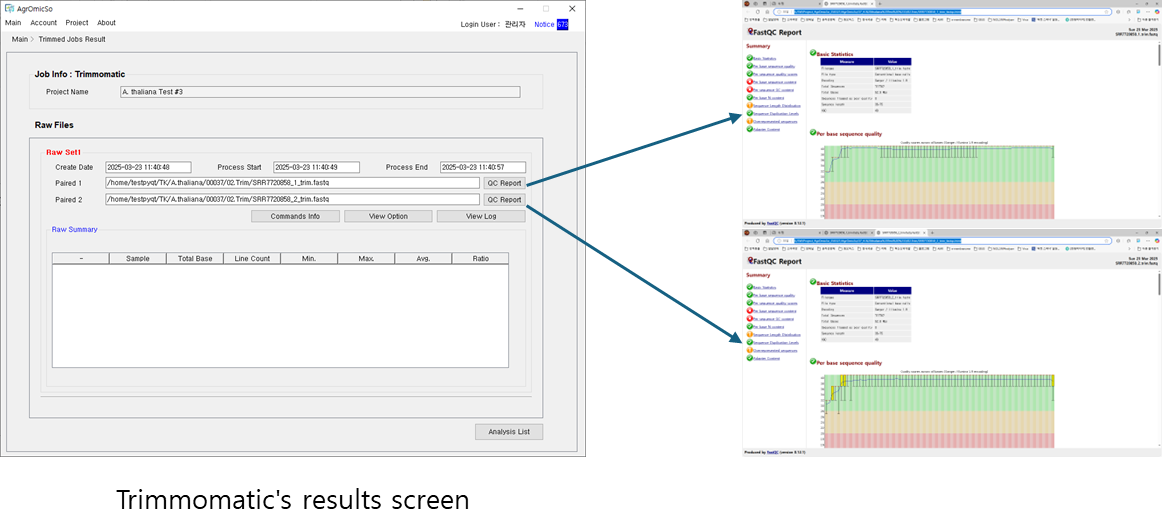
**

**Slide 16**

# AgrOmicSo™ How to Use - 7. Run Mapping

- The process of mapping NGS data to a reference sequence.
- The reference sequence and genetic information (GFF) are selected on the project creation screen (cannot be changed during the mapping step).

**
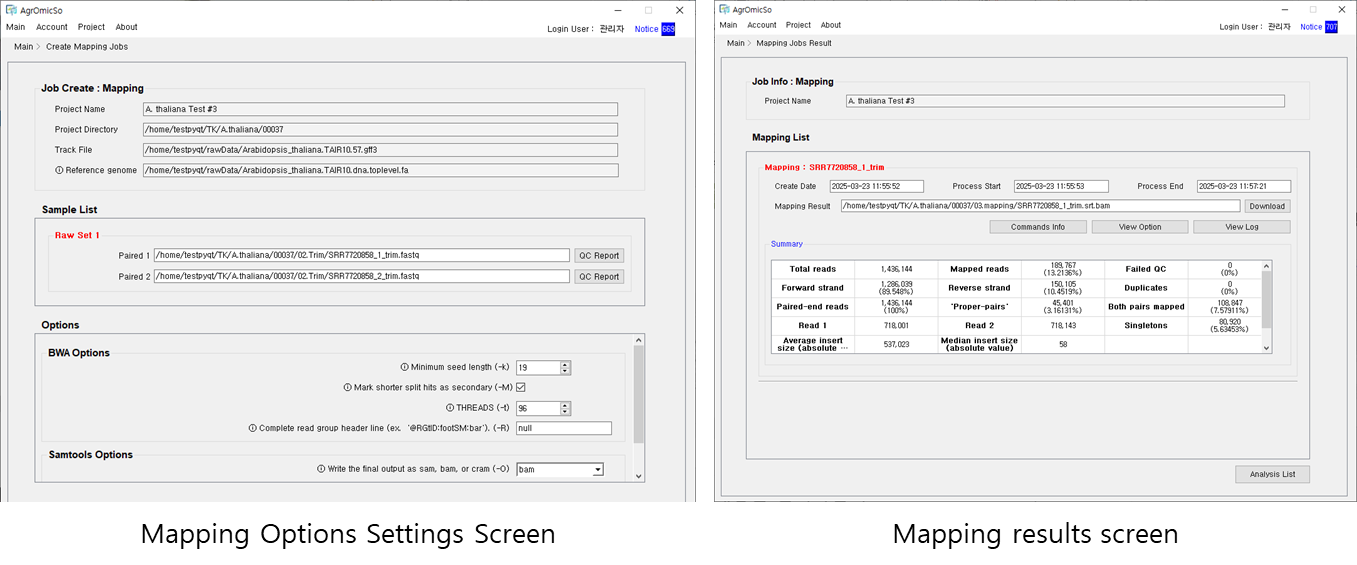
**

**Slide 17**

# AgrOmicSo™ How to Use - 8. Run Fixmate / Check Results

- Mate information in NGS data refers to information about sequenced read pairs.
- A function to correct mismatches in mate information.

**
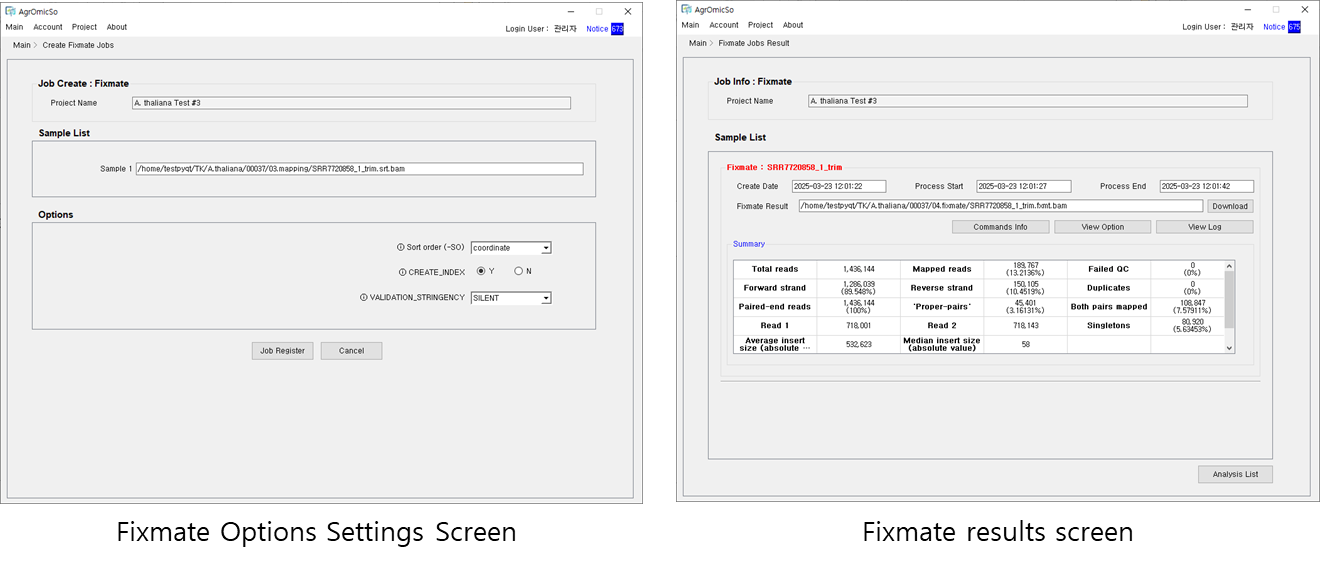
**

**Slide 18**

# AgrOmicSo™ How to Use - 9. Run Filter

- Unmapped leads
- Mismatched mate information
- Low-quality leads
- Duplicate leads

**
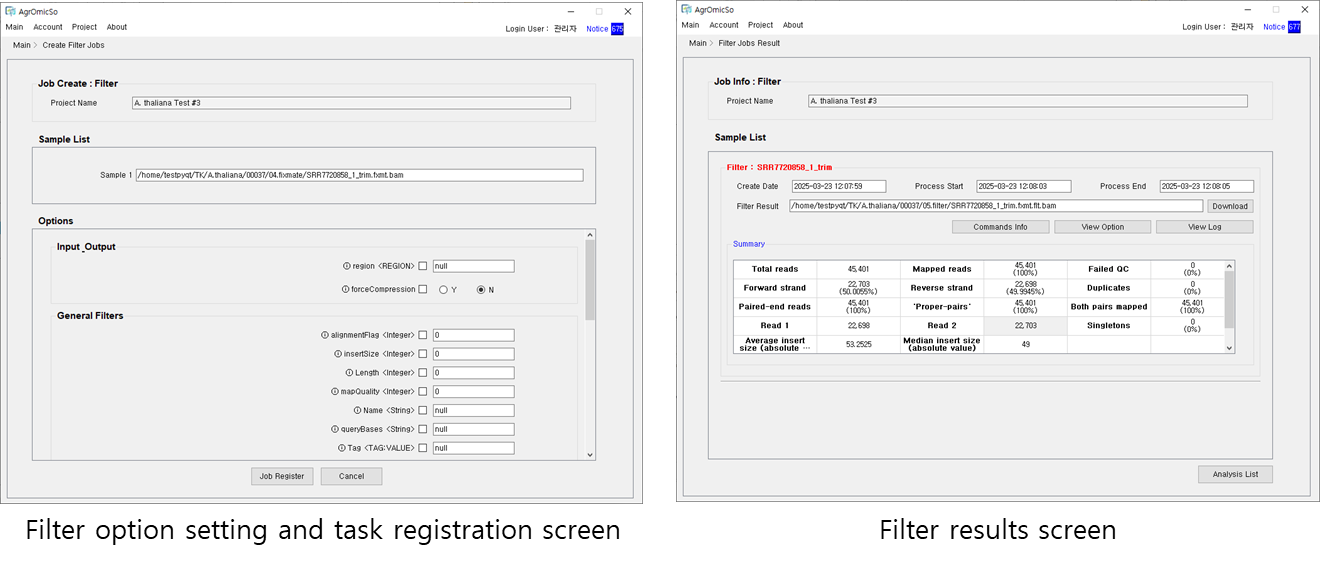
**

**Slide 19**

# AgrOmicSo™ How to Use - 10. Run Remove Duplicates

- Reads starting at the same location are considered duplicates, and these duplicate reads are marked and excluded from variant analysis.

**
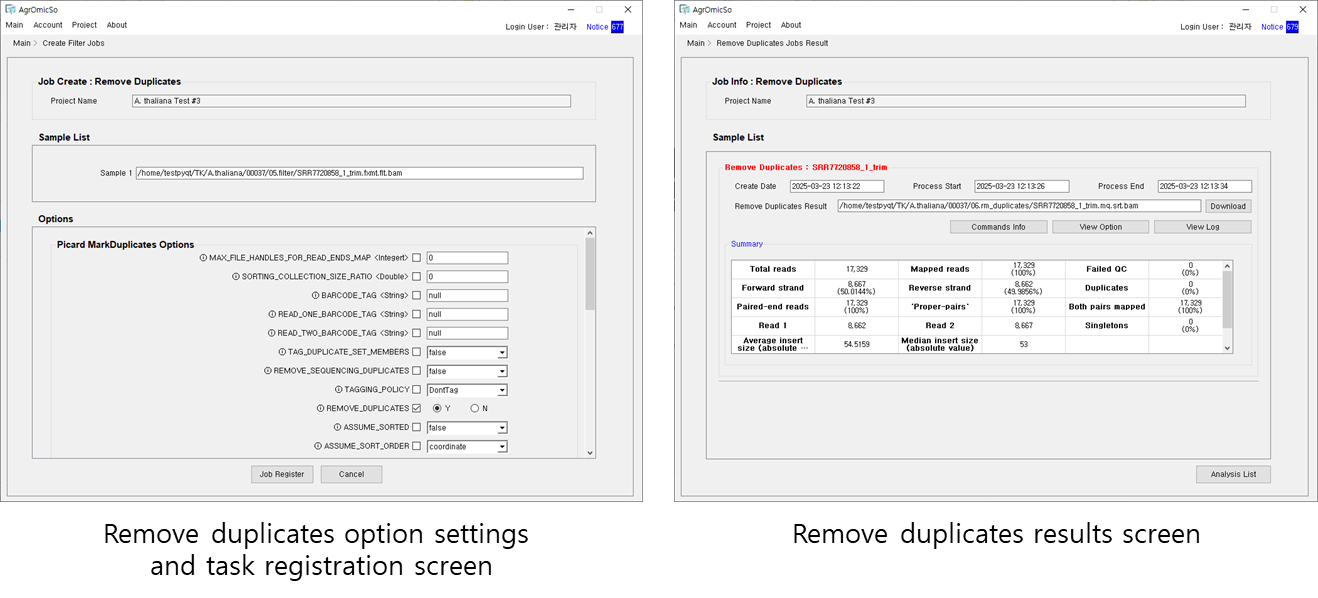
**

**Slide 20**

# AgrOmicSo™ How to Use - 11. Run Variant Call (GATK)

- GATK (Genome Analysis Toolkit) is a software tool for genomic data analysis, mainly used to perform genomic data analysis tasks such as variant calling.

**
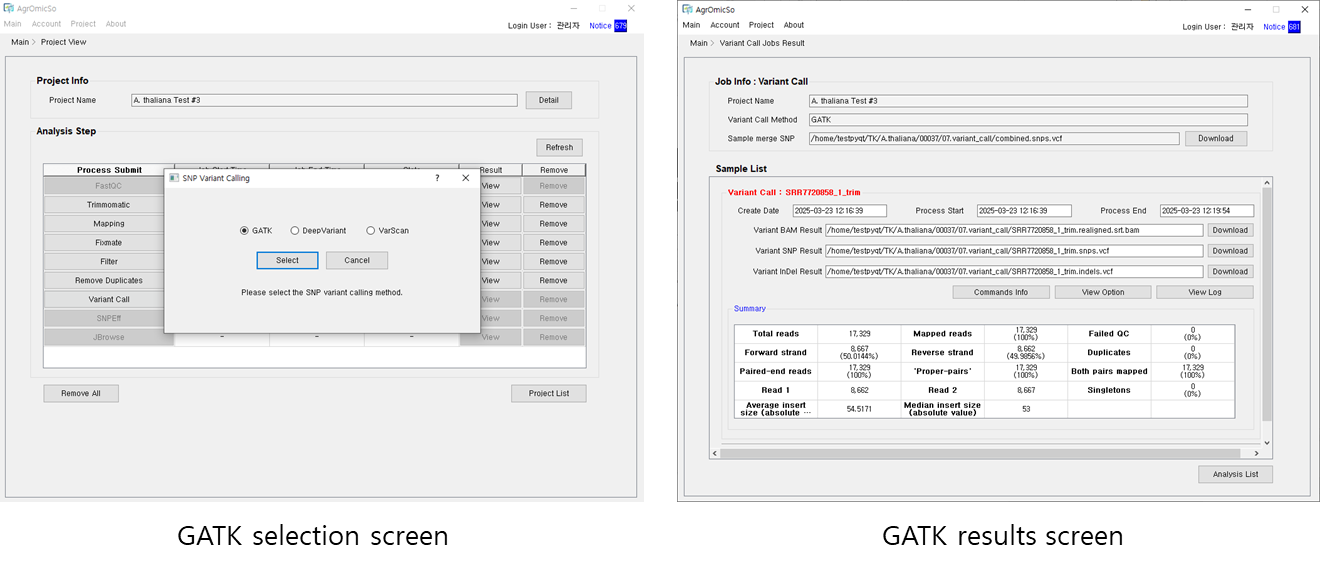
**

**Slide 21**

# AgrOmicSo™ How to Use - 12. Run Variant Call (DeepVariant)

- DeepVariant is a machine learning-based variant analysis tool developed by Google. It boasts high variant calling accuracy in NGS data.

**
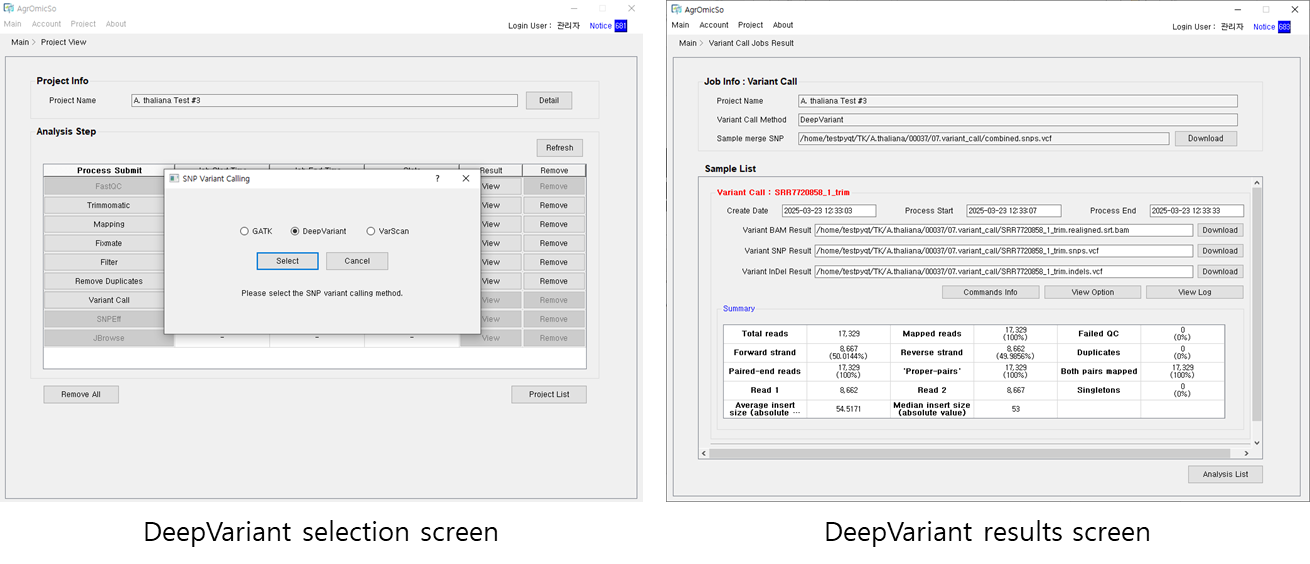
**

**Slide 22**

# AgrOmicSo™ How to Use - 13. Run Variant Call (VarScan)

- VarScan is a tool for analyzing mutations in NGS data.
- It detects mutations with high sensitivity, even in low-depth reads.

**
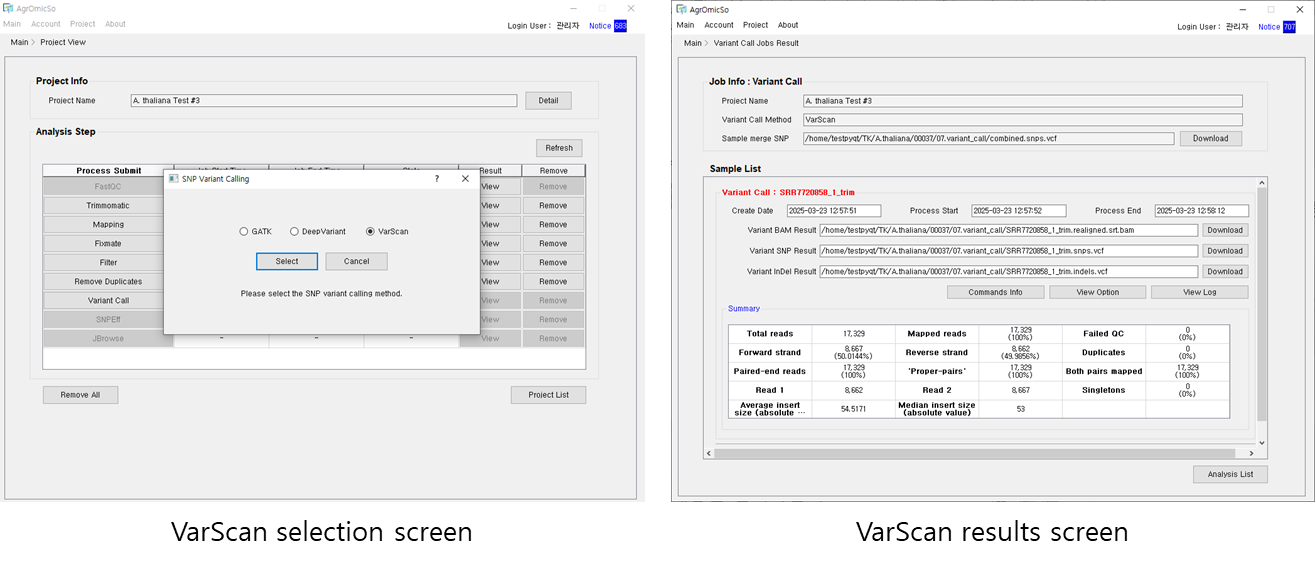
**

**Slide 23**

# AgrOmicSo™ How to Use - 14. Run SNPEff

- SNPEff is a tool for predicting the functional impact of mutations.
- It assesses the impact on a gene based on the mutation's location and type.

**
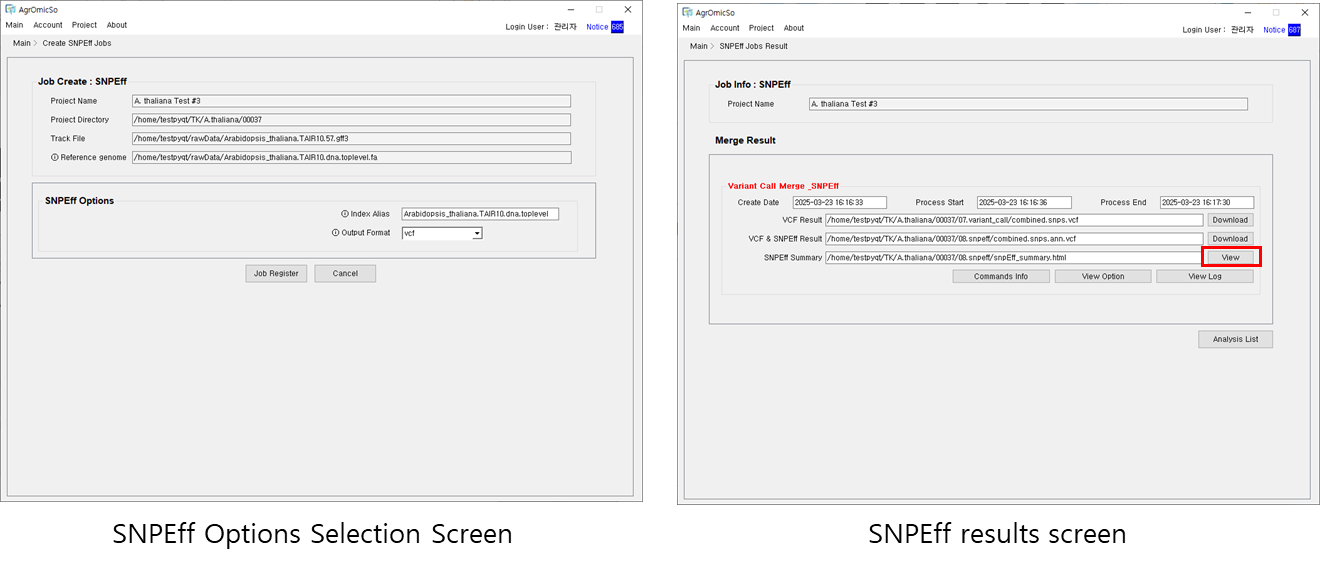
**

**Slide 24**

# AgrOmicSo™ How to Use - 14. Check SNPEff Results

- View button: Check the functional information of the variant predicted by SNPEff in a web browser.

**
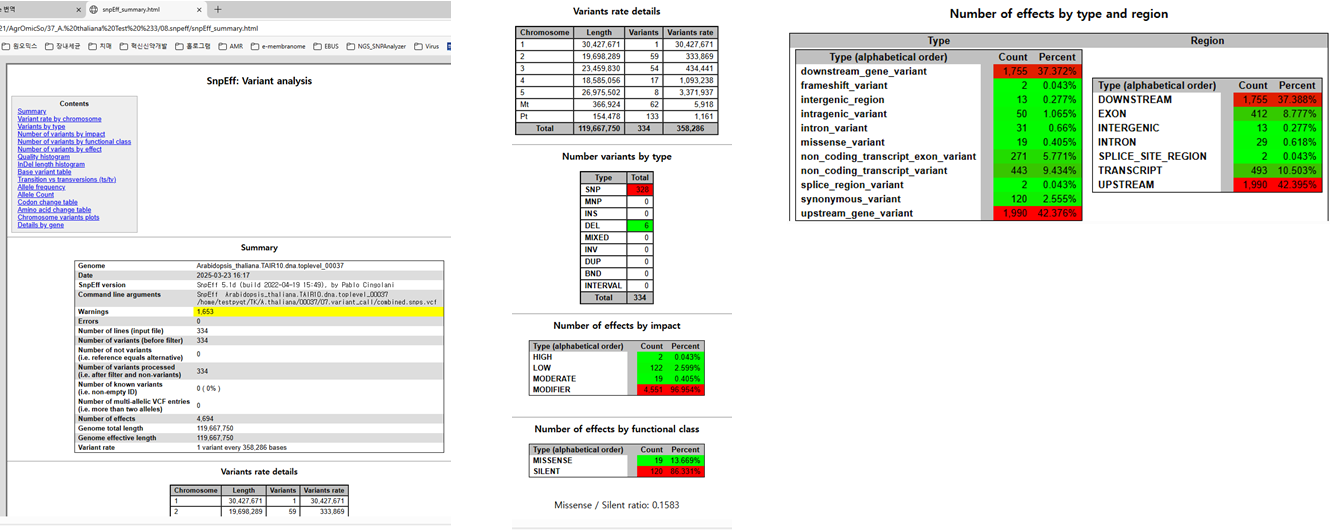
**

**Slide 25**

# AgrOmicSo™ How to Use - 15. Run JBrowse

- Visualization of variant analysis through the Variant Call step and function prediction results using SNPEff.

**
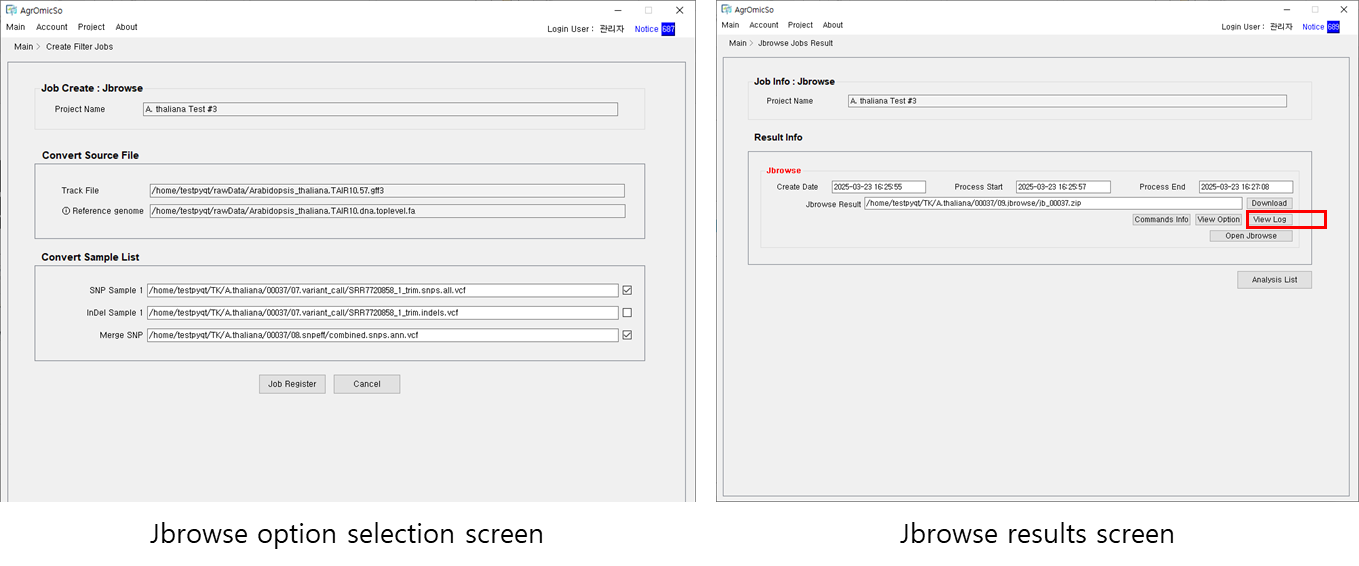
**

**Slide 26**

# AgrOmicSo™ How to Use - 15. Check JBrowse Results

- View and browse genomic variants visually using the JBrowse genome browser.

**
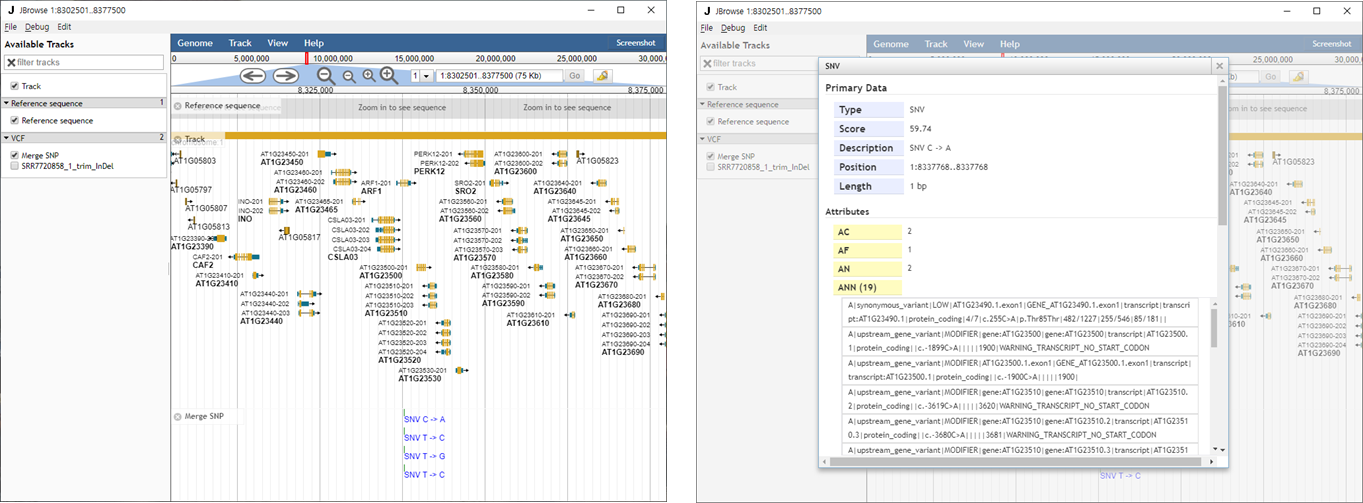
**

**Slide 27**

# AgrOmicSo™ How to Use - 16. Run One Step Process (Data Selection)

- This analysis method, which allows you to quickly proceed with the process of selecting and registering options step by step in a step-by-step process, by setting only the minimum number of options.
- [Data Selection]

**
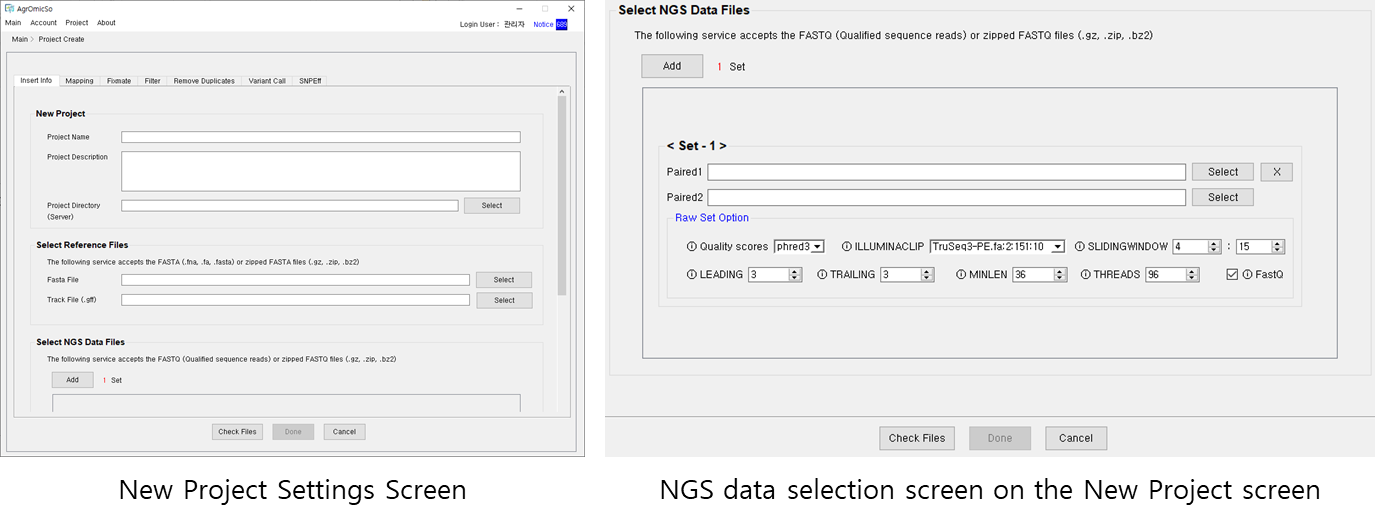
**

**Slide 28**

# AgrOmicSo™ How to Use - 16. One Step Process (Mapping & Fixmate Options)

- [Mapping Options]
- [Fixmate Options]

**
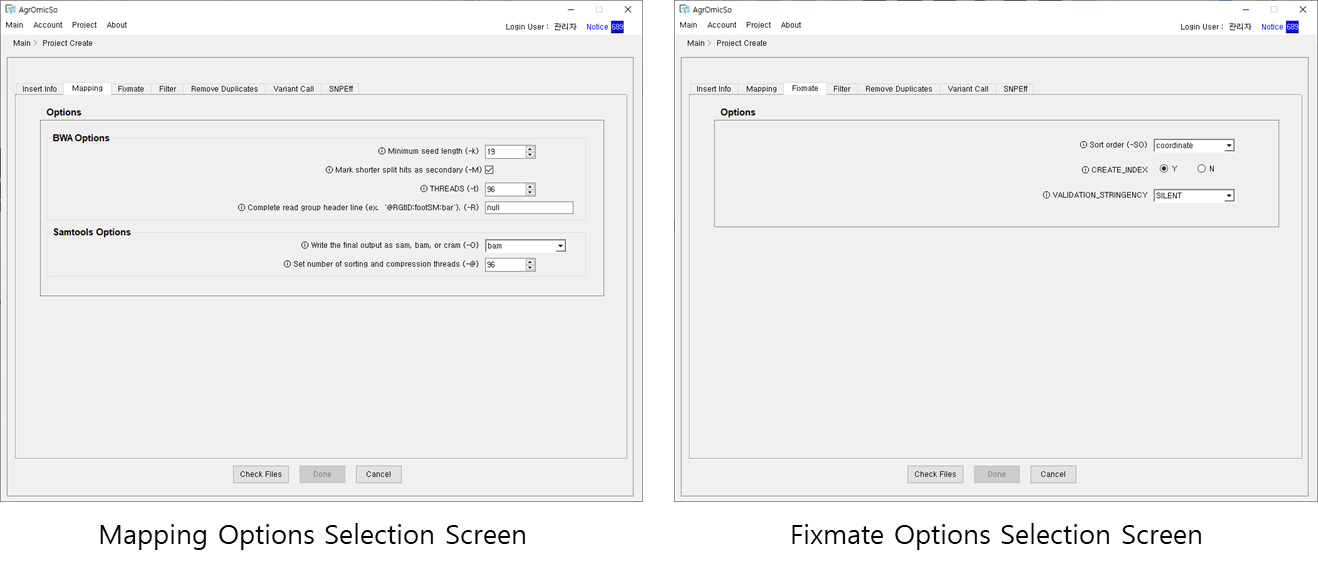
**

**Slide 29**

# AgrOmicSo™ How to Use - 16. One Step Process (Filter & Remove Duplicates Options)

- [Filter Options]
- [Remove Duplicates Options]

**
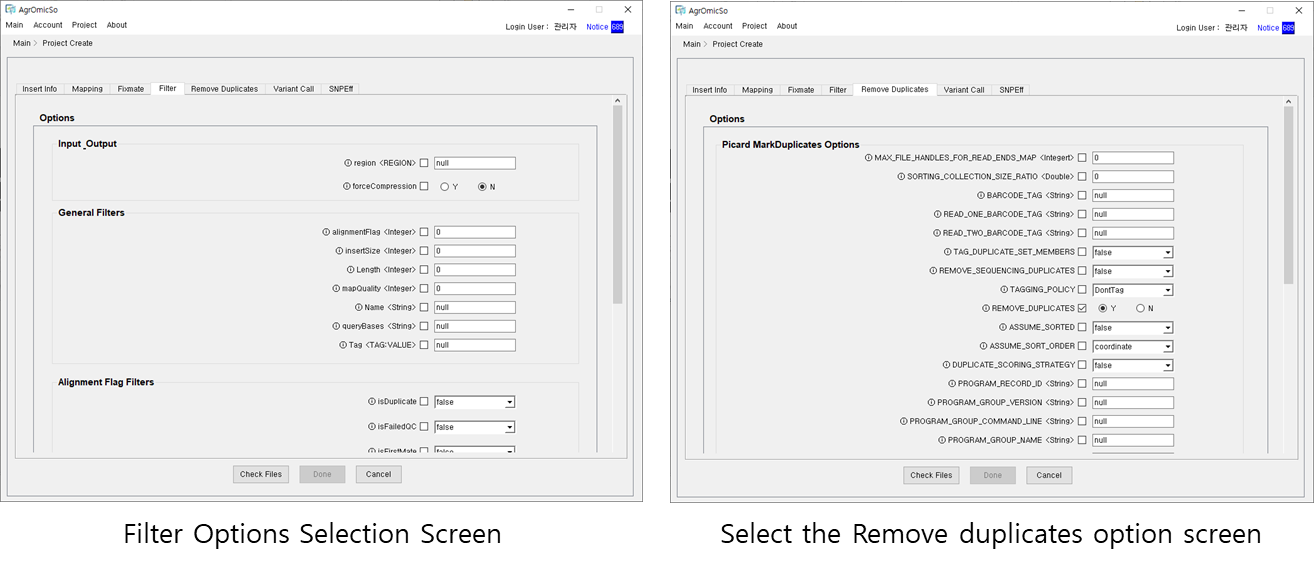
**

**Slide 30**

# AgrOmicSo™ How to Use - 16. One Step Process (Variant Call - GATK)

- [Variant Call Option - GATK]
- **
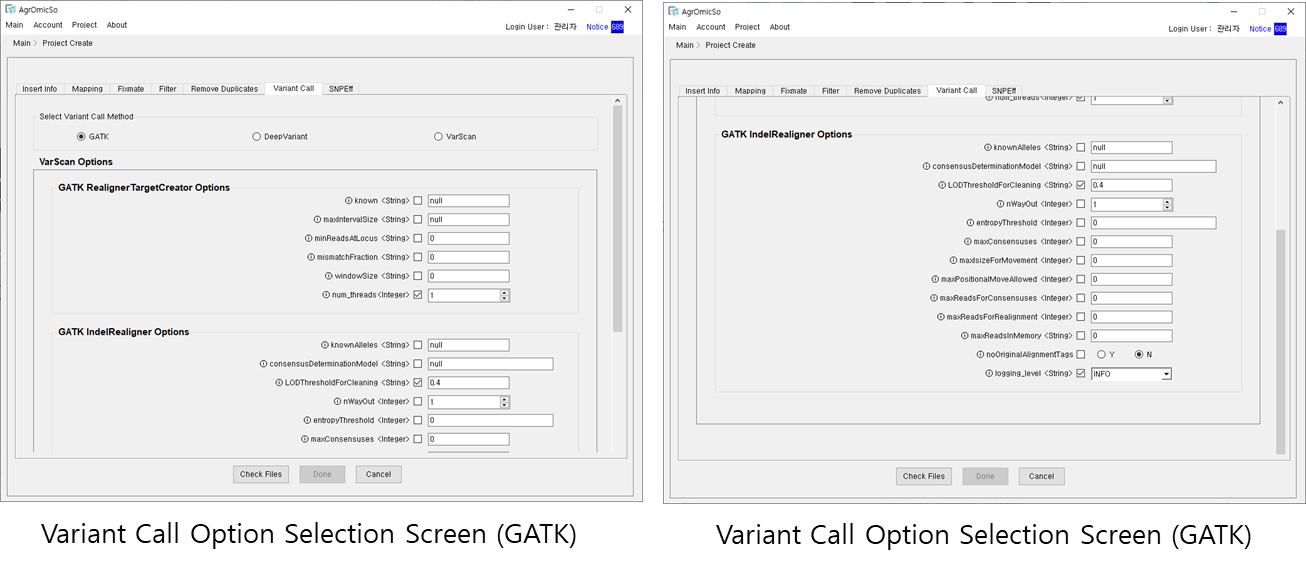
**

**Slide 31**

# AgrOmicSo™ How to Use - 16. One Step Process (Variant Call - DeepVariant & VarScan)

- [Variant Call Option - DeepVariant]
- [Variant Call Option - VarScan]

**
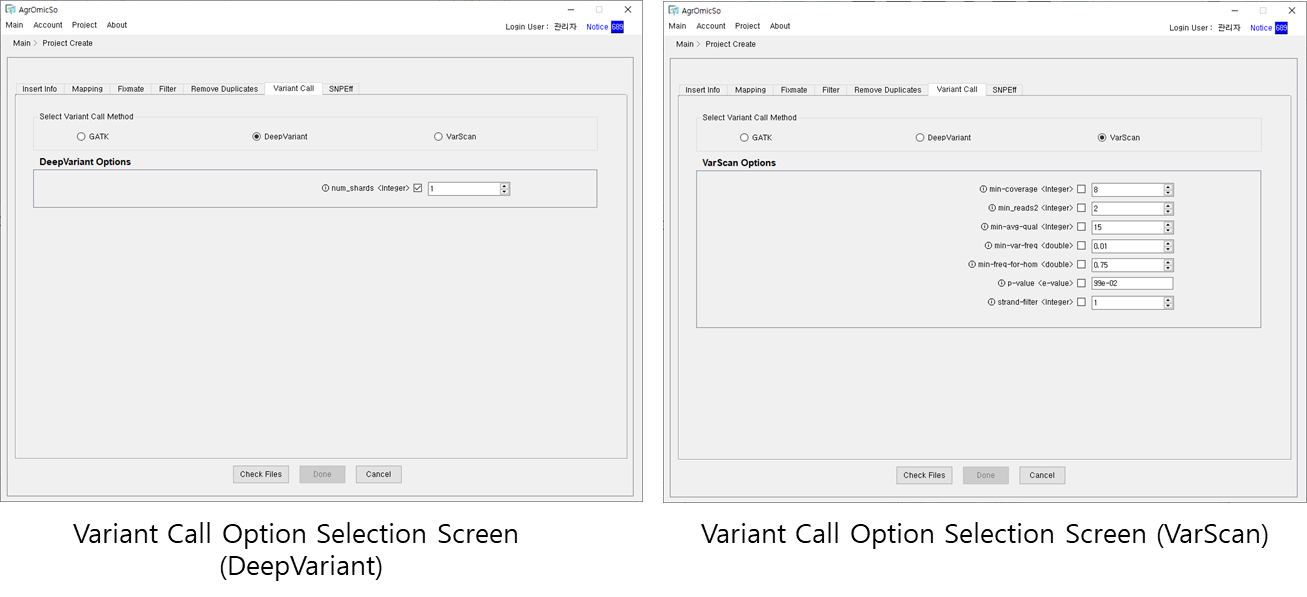
**

**Slide 32**

# AgrOmicSo™ How to Use - 16. One Step Process (SNPEff Option)

- [SNPEff option]

**
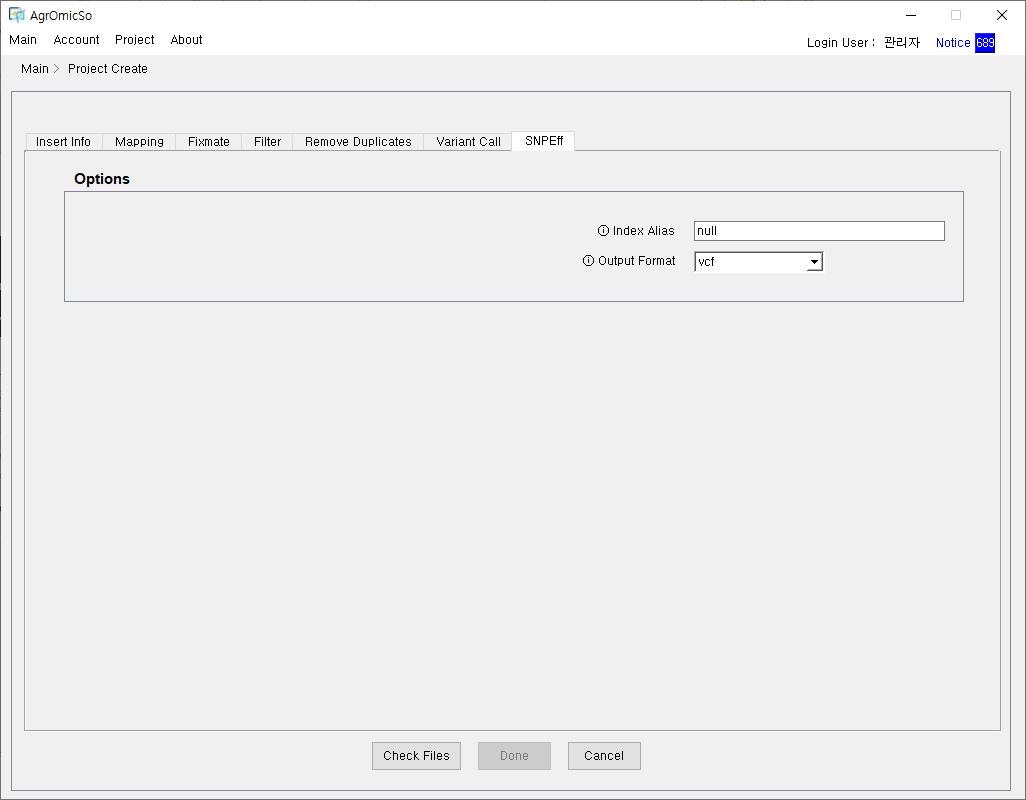
**

**Slide 33**

# AgrOmicSo™ How to Use - 16. One Step Process (Monitoring)

[Monitoring]

- Check the progress of your tasks on the same detailed screen as the Step-by-Step process.
- Unlike the Step-by-Step process, you can automatically move to the next step without having to register the next step.

**
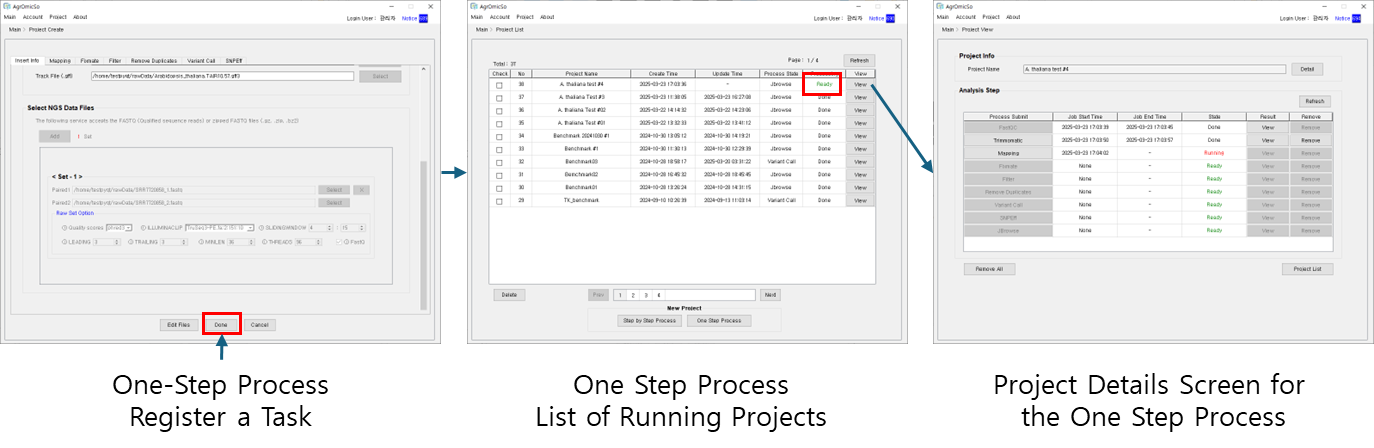
**

**Slide 34**

# AgrOmicSo™ How to Use - 17. Check JBrowse Results

Final visualization of analysis results in the JBrowse genome browser.

**
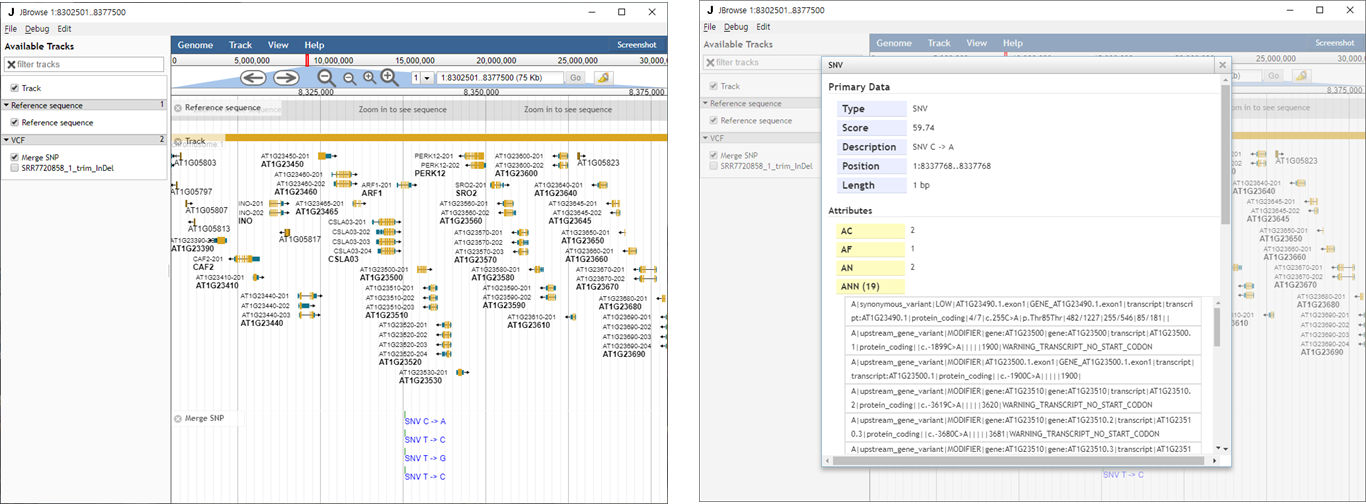
**
